# Integrative overview of antibodies against SARS-CoV-2 and their possible applications in COVID-19 prophylaxis and treatment

**DOI:** 10.1186/s12934-021-01576-5

**Published:** 2021-04-22

**Authors:** Norma A. Valdez-Cruz, Enrique García-Hernández, Clara Espitia, Laura Cobos-Marín, Claudia Altamirano, Carlos G. Bando-Campos, Luis F. Cofas-Vargas, Enrique W. Coronado-Aceves, Ricardo A. González-Hernández, Pablo Hernández-Peralta, Daniel Juárez-López, Paola A. Ortega-Portilla, Sara Restrepo-Pineda, Patricio Zelada-Cordero, Mauricio A. Trujillo-Roldán

**Affiliations:** 1 Programa de Investigación de Producción de Biomoléculas, Departamento de Biología Molecular y Biotecnología, Instituto de Investigaciones Biomédicas, Universidad Nacional Autónoma de México, Ciudad Universitaria, 04510 Ciudad de México, México; 2 Instituto de Química, Universidad Nacional Autónoma de México, Ciudad Universitaria, 04510 Ciudad de México, México; 3 Departamento de Inmunología, Instituto de Investigaciones Biomédicas, Universidad Nacional Autónoma de México, Ciudad Universitaria, 04510 Ciudad de México, México; 4 Facultad de Medicina Veterinaria Y Zootecnia, Universidad Nacional Autónoma de México, Ciudad Universitaria, 04510 Ciudad de México, México; 5 Escuela de Ingeniería Bioquímica, Pontificia Universidad Católica de Valparaíso, Av. Brasil N° 2950, Valparaíso, Chile

## Abstract

SARS-CoV-2 is a novel β-coronavirus that caused the COVID-19 pandemic disease, which spread rapidly, infecting more than 134 million people, and killing almost 2.9 million thus far. Based on the urgent need for therapeutic and prophylactic strategies, the identification and characterization of antibodies has been accelerated, since they have been fundamental in treating other viral diseases. Here, we summarized in an integrative manner the present understanding of the immune response and physiopathology caused by SARS-CoV-2, including the activation of the humoral immune response in SARS-CoV-2 infection and therefore, the synthesis of antibodies. Furthermore, we also discussed about the antibodies that can be generated in COVID-19 convalescent sera and their associated clinical studies, including a detailed characterization of a variety of human antibodies and identification of antibodies from other sources, which have powerful neutralizing capacities. Accordingly, the development of effective treatments to mitigate COVID-19 is expected. Finally, we reviewed the challenges faced in producing potential therapeutic antibodies and nanobodies by cell factories at an industrial level while ensuring their quality, efficacy, and safety.

**Supplementary Information:**

The online version contains supplementary material available at 10.1186/s12934-021-01576-5.

## Introduction

The recent disease outbreak caused by the new severe acute respiratory syndrome coronavirus 2 (SARS-CoV-2) is a global health emergency, as april 2021 affecting more than 134 million people and leading to almost 2.9 million deaths until date [1]. In the last two decades, other SARS-CoV-2-related pathogenic β-coronaviruses have caused syndromes such as severe acute respiratory syndrome (SARS-CoV) and Middle East respiratory syndrome (MERS-CoV). SARS-CoV-2 is the causative agent of coronavirus disease (COVID-19). As the most transmissible coronavirus (CoV) identified to date, its vertiginous spread has led to the current COVID-19 pandemic [2–5]. This emphasizes the urgency in the research, design, innovation, and large-scale production of new prophylactic and therapeutic drugs.

CoVs are enveloped single-stranded positive-sense RNA viruses that can infect an extensive number of hosts. Human CoVs (order Nidovirales, family Coronaviridae, subfamily Coronavirinae) are zoonotic pathogens, i.e., they can infect humans via interspecies transmission [6–8]. SARS-CoV-2 is a β-coronavirus that has four structural proteins: the nucleocapsid (N), membrane (M), envelope (E), and surface-anchored spike glycoprotein (S), which is proteolytically processed, generating a trimmer with three S_1_ subunit heads sitting on top of a trimeric S_2_ subunit, that allow the subsequent virus fusion [9–11]. The S protein, through its three receptor binding domains (RBDs), interacts with the human angiotensin-converting enzyme (hACE2), as an entry receptor, and the S_2_ subunit induces fusion to the cell membrane [10, 12–15]. For this reason, the S protein represents an interesting target for the rational production of vaccines or therapeutic antibodies (Abs) preventing infection [16–18]. Due to the extensive transmission worldwide, the genetic diversity of the virus is dynamic. Recurrent mutations may indicate a convergent evolution for adaptation in humans, similar to those occurring in the S protein [19].

Since the beginning of the SARS-CoV-2 infectious outbreak, diverse antiviral chemical compounds have been tested in the clinic, showing different efficacies. For instance, the antiviral remdesivir is authorized in the United States for emergency use in humans [20, 21], although some trials do not show substantive benefits [22]. Moreover, approximately 180 vaccine candidates are under development awaiting expedited approval, upon demonstration of proof of quality, safety, and efficacy. More than four different vaccines are approved for emergency use by the Food and Drug Administration (FDA) and other regulatory agencies [23–25]. In addition, various recombinant monoclonal antibodies (mAbs) are being tested in therapy, with targets such as C5a, IL-6, and PD-1, among others, to curb some of the responses caused by SARS-CoV-2 [23]. Similarly, various mAbs developed and tested against other CoVs, including SARS-CoV and MERS-CoV, have been tested against SARS-CoV-2 to treat COVID-19 [3, 12, 26–30]. Alternatively, the World Health Organization recommends the use of plasma from convalescent patients as a therapy to treat critically ill patients globally [5, 31, 32]. Therefore, there is a need for the development of effective and safe COVID-19-specific vaccines or therapeutic drugs. Neutralizing Abs are one of the best candidates for neutralizing virus infection due to their antigenic specificity [12, 29, 30, 32]. Artificial passive immunization was born as a therapy based on antibodies transference from serum of immunized animals or humans to a recipient, conferring an immune state against the target [33]. Furthermore, this is one of the most employed immunotherapies in medicine history, supported by a long list of uses based on its neutralization activities upon infectious diseases as produced by bacterial toxins as *Corynebacterium diphteriae* [34], *Clostridium tetani* [35], *Staphylococcus aureus* [36], *Clostridium dificile* [37], *Bordetella pertussis* [38], among others. Also, successful viral neutralization had been described such as Enterovirus [39], Hepatitis B virus [40], Measles virus [41], Parvovirus [42], Rabies virus [43], Respiratory syncytial virus (RSV) [44] and Varicella–zoster virus [45]. Nowadays, there is technology to produce monoclonal high-specific and long-lasting antibodies from in vitro systems [46], which means a relevant therapeutic weapon to fight a wide spectrum of infections and other pathologies. Whilst active immunization by infection or vaccination requires a period of time to generate its own system antibodies, passive immunity represents an instantly effective source which induces immunological events as neutralization, opsonization, complement activation and antibody dependent cellular cytotoxicity (ADCC). Furthermore, passive immunity does not depend on recipient immune response which implies a critical instrument to treat immunocompromised patients and other vulnerable groups who cannot be exposed to the antigen itself. In front of COVID-19 pandemic, antibody-based humoral passive immunization treatment is a promising route to treat severe cases, or people who do not respond to vaccination or cannot be vaccinated.

There are several reviews on specific aspects of Abs or its formats as alternative treatments for COVID-19 [47–51]. Moreover, information about Abs is updated daily and is tremendously enriched, then different public databases have compiled information, allowing quick searches [52, 53]. Here, we update the knowledge regarding the immune response associated with COVID-19, the formation of neutralizing Abs towards SARS-CoV-2 in the plasma of patients, which could be useful in prophylactic and therapeutic treatments. Moreover, we discuss on the development and isolation of Abs from different sources (hybridomas production, the generation of nanobodies, and the recombinant production of fully humanized mAbs) against different SARS-CoV-2 targets, in an integrative form. This review also incorporates a comprehensive view of the challenges that faces the cell factories at an industrial level to produce therapeutic Abs and their formats, guaranteeing the corresponding quality, efficacy and safety attributes in the bioprocess. Due to the importance of certain references, 18 preprints were considered.

## The immune response and physiopathology of COVID-19

COVID-19 is highly contagious, and oral-respiratory droplet contamination as well as aerosols, have been implicated in its transmission [54, 55]. A wide spectrum of associated clinical symptoms have been described, such as gastrointestinal issues, diarrhea, shortness of breath, headache, sore throat, cold, breathing difficulties, myalgia, nasal congestion and inflammation of the mucous membranes, and central nervous system injuries [56], and patients present with asymptomatic to severe infections, or even succumb to the disease [57]. There is an important relationship between the COVID-19 severity and activation/suppression of the immune response elements. SARS-CoV-2 is a virus with the ability to generate an acute and destructive inflammatory response affecting the tissues and various cell types that express the hACE2 receptor [10, 58, 59]. hACE2 is a type I membrane protein present in the human organs, including the lungs, heart, kidney, and intestine [60]. The inflammatory response is induced by different immunological mechanisms associated with the innate and adaptive immune responses.

### Innate immune response against SARS-CoV-2 infection

**Fig. 1:**
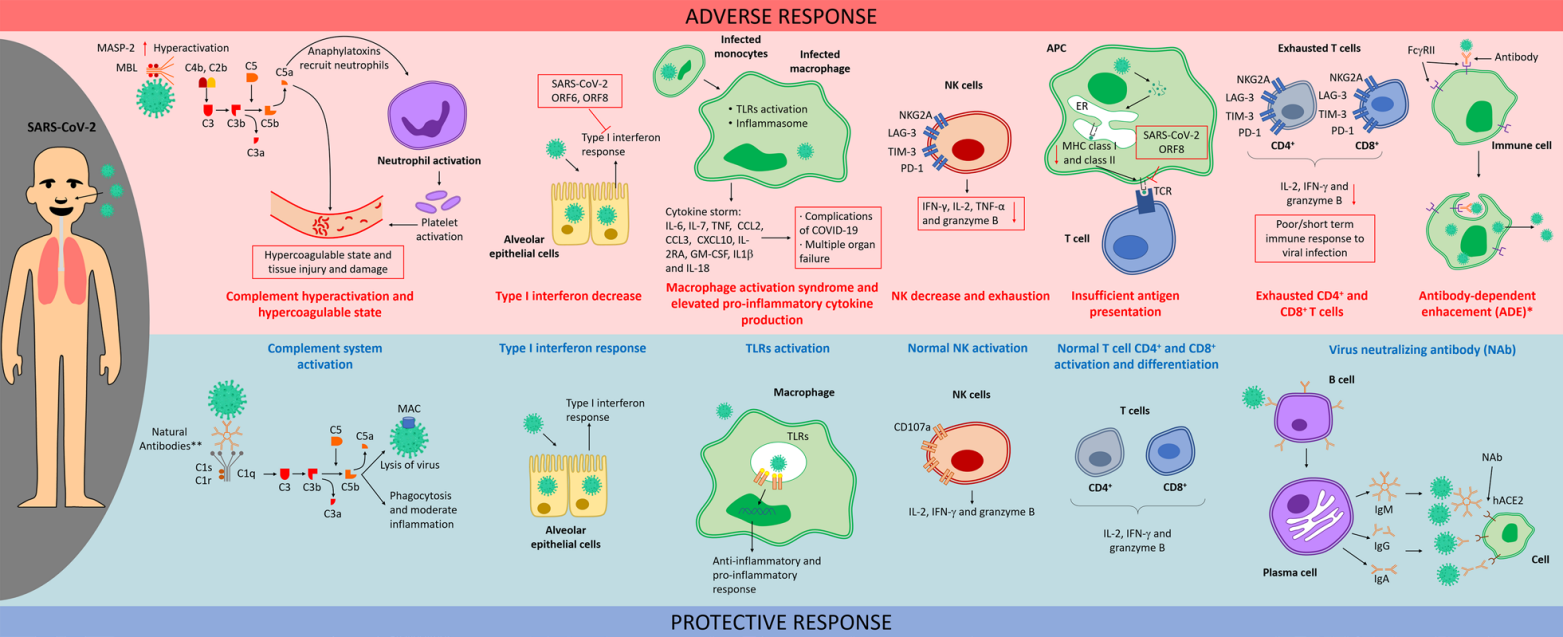
Mechanisms in adverse and protective immune response for SARS-CoV-2. Upper panel (red). Adverse immune response in the presence of SARS-CoV-2 include mechanisms like complement hyperactivation and hypercoagulable state, excessive macrophage migration, macrophage activation syndrome, NK exhaustion, insufficient antigen presentation, exhausted CD4^+^ and CD8^+^ T cell and antibody-dependent enhancement (ADE) *This response has been described by in vitro models. Lower panel (blue). Protective immune response is characterized by complement system activation trough IgM natural antibodies (** this has been suggested as an initial barrier for SARS-CoV-2 infection), TLRs activation, NK and T cell normal activation and antibody virus neutralization by B cells. *APC* antigen presenting cell, *ER* endoplasmic reticulum, *FcgRII* receptor II for the Fc region of immunoglobulin G, *GM-CSF* Granulocyte–macrophage colony-stimulating factor, *MAC* membrane attack complex, *MBL* mannan-binding lectin, *MHC* major histocompatibility complex, *MSP* mannose-associated serine proteases, *Nab* neutralizing antibody, *TCR* T-cell receptor, *TLR* Toll-like receptor

The innate immune response to SARS-CoV-2 is characterized by not only the activation of epithelial cells, but also the hyperactivity of macrophages. In the presence of the virus, macrophages and respiratory epithelial cells have the ability to release proinflammatory and inflammatory mediators by activating the inflammasome, and pattern recognition receptors induced by virus pathogen-associated molecular patterns (PAMPs) [61]. RNA from different viruses, such as CoVs, acts as a PAMP that can be detected by various toll-like receptors (TLRs), such as TLR3, TLR7, TLR8, and TLR9 [62], activating the nuclear factor kappa light chain enhancer of activated B cells (NF-κB) pathway and proinflammatory cytokines [61]. During COVID-19 infection, it has been observed that monocytes have a relevant contribution in the progression of the disease towards severe manifestations, because the systemic profiles of cytokines in patients are similar to that in some of the syndromes, such as macrophage activation syndrome [63], or cytokine storm [64, 65]. This immune response is related to a high production of cytokines (IL-6, IL-7, and TNF-α) and inflammatory chemokines, including CCL2, CCL3, and CXCL10, as well as the IL-2 receptor α-chain in the soluble form [64, 66]. Multiple organ failures and complications such as acute respiratory distress syndrome (ARDS), which could cause the death in patients (Fig. 1, Additional file 1: Table S1), have been related with the systemic overproduction of cytokines [65, 67].

The role of macrophages can be deduced from the immune response noted in other CoV infections, such as that for SARS-CoV, which has an accessory protein open reading frame 8 (ORF8) that activates a family of PAMPs called nucleotide-binding domain and leucine-rich repeat pyrin domain 3 (NLRP3) [68]. NLRP3 can form multiprotein complexes, termed “inflammasomes” that activate caspase-1, which leads to the maturation of proinflammatory cytokines (IL-1β and IL-18), and induction of pyroptosis [69]. Moreover, this protein is present in SARS-CoV-2, and although its participation in the immune response has not been described, it is likely that NLRP3 could be associated with the aberrant activation of macrophages and elevated levels of IL-1β and IL-18 in some of the patients with COVID-19 [70] (Fig. 1 and Additional file 1: Table S1).

Forty-two percent of patients with pneumonia due to COVID-19 present severe ARDS [71]. This is reflected by the macrophage infiltration in the lung tissues observed *post mortem* [72]. Several studies have reported that macrophage hyperactivation results in pathological effects; this led us to hypothesize that a balance between anti-inflammatory and proinflammatory activities may be related to a protective immune response (Fig. 1 and Additional file 1: Table S1).

In severe cases, there is a decrease in natural killer (NK) cell populations [73]. Moreover, in patients infected with SARS-CoV-2, their NK cell population have shown lower percentages of intracellular CD107a, IFN-γ, IL-2, TNF-α, and granzyme B compared with that in healthy subjects, and an exhaustive phenotype characterized by the overexpression of NK group 2 member A (NKG2A) [74]. An inhibitory receptor related to the dysfunctional NK cell phenotype [75] has also been observed in chronic viral infections [74, 76] as well as in COVID-19 patients with other NK cell exhaustive phenotype molecules, such as lymphocyte-activation gene-3 (LAG-3), programmed cell death protein 1 (PD-1), mucin domain-3 (TIM-3), and T-cell immunoglobulin [77] (Fig. 1 and Additional file 1: Table S1).

Another element of the innate immune response participating in COVID-19 pathophysiology is the complement system, which can be activated by an antibody-independent mechanism, termed the “lectin pathway”. This mechanism uses, among other proteins, mannan-binding lectin-associated serine protease 2 (MASP-2), which can generate fragments of complement components, such as C5a that are potent mediators of inflammation and chemoattractants for neutrophils and monocytes. Since the SARS-CoV-2N protein can activate MASP-2 [78], it may lead to the hyperactivation of the complement system that can cause significant damage, specifically damage related to neutrophil migration and activation in the lung tissues [79], and lead to hypercoagulation, as observed in critical patients [78]. Proteins of the complement system can also participate in coagulation [80] (Fig. 1 and Additional file 1: Table S1). Remarkably, MASPs have been shown to cleave prothrombin into thrombin [81]. The C5a receptor in neutrophils leads to the induction of the blood coagulation cascade [82], and C5b-9 stimulates procoagulant activity through platelet prothrombinase [83]. Some studies have suggested the potential SARS-CoV-2-specific antiviral effects of natural IgM Abs against A blood group produced by B1, in a complement-dependent manner, thereby proposing natural Abs as an initial barrier to infection and speculating a relationship between the reduced antibody diversity present in older patients [84] with severe illness [85] (Fig. 1 and Additional file 1: Table S1).

It has been suggested in different animal models infected with other viruses that an acute lung injury can be caused due to monocyte activation through mechanisms that could occur in SARS-CoV-2 infection. For example, viruses such as H5N1 avian influenza and SARS-CoV can activate macrophages by oxidative stress in a murine model [86, 87]; IgG anti-SARS-CoV S protein immune complexes can polarize the macrophage response into an inflammatory response in macaques [88].

Type I and III IFNs can control viral infection [89], but delayed interferon signaling in SARS-CoV-2 infection is related to robust virus replication and severe complications [90]. The decrease in IFN production is associated with ORF6, ORF8, and nucleocapsid proteins that inhibit the type I IFN signaling pathway [70] (Fig. 1 and Additional file 1: Table S1).

### Effective adaptive immune response against SARS-CoV-2 infection

In COVID-19 the immune response associated with lymphocytes present heterogeneity as human diversity, but in many cases correlates with the severity of the disease. In adaptive cellular immune response patients with COVID-19 show a dramatic reduction in total T cells, which is negatively related to patient survival; these T cells express exhaustive signatures, such as PD-1, TIM-3, and LAG-3 [91, 92], all of which are immune-inhibitory factors [93, 94]. Evidence shows that CD8^+^T numbers are low in patients with severe COVID-19 compared with less severe cases [95, 96]. CD8^+^ T cells also express exhaustive-type cell phenotypes similar to NK cells (high expression of NKG2A and low expression of intracellular CD107a, IFN-γ, IL-2, TNF-α, and granzyme B+) [74, 97]. In addition, the T_reg_ and CD4^+^T memory lymphocyte counts are reduced [56, 73, 96]. In the same sense, older patients with some comorbidity had a higher number of activated virus-specific CD4^+^T cells compared to patients who had fewer risk factors. Moreover, these cells show an increase in IL-2 secretion and a diminishing in the IFN-γ production [98]. Furthermore, lymphopenia has been associated with an increase in mortality [99], this probably caused by the infection of SARS-COV-2 to lymphocytes, which express hACE2 [100]. In addition, exhaustion of lymphocytes, has been observed in severe cases [101]. While in mild disease, an increased number of active CD8^+^T cells and greater clonal expansion has been observed [101], as well as more IFN-γ-producing T helper 1 (TH1) cells. Notably, in recovered patients a strong memory T cell response in peripheral blood has been detected, being wider and intense in patients with severe condition compared to mild cases [102]. As well as the COVID-19 recovered patients have virus-specific memory CD4^+^T and CD8^+^T cells [103], which could be an indicative of protective immunity (Fig. 1, Additional file 1: Table S1).

Other cell populations, such as plasmacytoid dendritic cells and γδ T cells, have been reported to be almost depleted in SARS-CoV-2 infection [77]. Regarding antigen (Ag) presentation for T-cell activation, the ORF8 protein of SARS-CoV-2 can interact with the major histocompatibility complex class I (MHC-I) molecule, causing its downregulation and provoking the conjugate internalization to lysosome for its further degradation, avoiding the Ag presentation, being proposed as a via of immune evasion through ORF8 [104]. Furthermore, there is also evidence of downregulation of at least eight genes encoding MHC-II molecules in peripheral monocytes isolated from ventilation-dependent patients, relative to that in healthy subjects [77] (Fig. 1 and Additional file 1: Table S1). In contrast, the humoral arm of the adaptive response may facilitate, on rare occasions, the entry of viruses into host cells and enhancement of viral infection by a process independent of their specific cell receptors, known as “antibody-dependent enhancement (ADE)” [105, 106]. ADE comprises the production of sub-neutralizing or non-neutralizing Abs with a paradoxical effect associated with the virus-antibody interaction, with Fc receptors on different immune cells improving viral infection and replication [107, 108]. This event has been described to be related to other CoVs, such as MERS-CoV, SARS-CoV, and feline CoVs [88–112]. However, in the case of SARS-CoV-2, this association has not been demonstrated in patients [113]. Nevertheless, there is evidence from in vitro models that show ADE promoted by Abs isolated from severely affected patients’ plasma, relating to the FcγRII engagement [114] (Fig. 1 and Additional file 1: Table S1). Hence, ADE should be monitored in vaccination or therapeutic strategies against SARS-CoV-2 infection.

The controversy about the exacerbation of the disease and the appropriate response to resolve COVID-19 is still under discussion. However, hospital patients coincide in an insufficient or excessive immune response, compared to those individuals without serious consequences. This remarks that the set of innate and adaptive responses and their balance is important for a favorable progression, being highlighted that the humoral immune response points out that specialized neutralizing antibodies are the most important molecules for the protection against infection.

#### Abs and their isotypes

Dating back to the 1790s, Abs have been described as a protective substance in the serum after vaccination [115, 116]. Abs are used by the immune system to identify and neutralize elements, such as bacteria and viruses [117]. Abs are composed of proteins (82–96%) and carbohydrates (4–18%), and are divided into five immunoglobulin isotypes (IgG, IgA, IgM, IgE, and IgD), which differ in structure, abundance/distribution, specificity, and half-life [118].

Seric IgA is present in the plasma, and its secreted form (sIgA) is present in the mucous membrane, tears, and saliva, which prevents the colonization of pathogens in the respiratory, gastrointestinal, and urogenital tracts [119]. The sIgA is capable of inducing the synthesis of IL-6, IL-8, monocyte chemoattractant protein-1 (MCP-1), and the granulocyte–macrophage colony-stimulating factor (GM-CSF) in the lung fibroblasts [120], which leads to hypothesizing its participation in severe cases of COVID-19 [121]. IgD is an antigen receptor localized at the surface of different B-cells, and its expression is balanced with IgM depending on the antigens sensed [122, 123]. The secreted IgD is produced by mucosal B cells such as plasmablasts or plasma cells and improves mucosal homeostasis and prepares basophils and mast cells to protect the system against antigens, producing cytokines [122]. IgE has antiparasitic activity and responds to allergens, releasing histamine from mast cells and basophils [124]. IgM is also an Ag receptor in B cells and is the first to be secreted during the primary humoral immune response, before IgG synthesis [117, 124]. IgG is the most prevalent isotype, specialized to recognize and neutralize Ags [125].

#### Humoral response

It is well recognized that the neutralizing humoral immune response is the main mechanism for preventing viral infections [126]. Particularly, antibody-mediated responses against SARS-CoV-2, as well as their kinetics have been described in COVID-19 patients. The appearance of IgM, IgA, and IgG that recognize SARS-CoV-2 has been determined [127]. The seroconversion of patients with COVID-19 is attained following the onset of symptoms, producing IgM, IgA, and IgG [127, 128]. IgM accumulation is observed within 7 days post symptom onset (PSO), which is useful as a marker of acute infection. In contrast, IgA titer increases principally between 8 and 21 days PSO [127]. Importantly, the median time of IgG appearance has been recorded as 14 days PSO [127]. Hence, the detection of anti-SARS-CoV-2 Abs IgM and IgG is a diagnostic. However, the IgG and IgM levels are found to be widely variable, and no correlation between the Ab titers and clinical characteristics of the patients has been found [128].

The response of serum IgA against the S protein is detectable from 6 to 8 days PSO, with a mean period of 13 days PSO [121], followed by attainment of a peak on days 20–22 and maintenance for at least 40 days [129]. Furthermore, patients with COVID-19 establish the seroconversion of IgM and IgG that recognize mainly N and S (RBD) proteins, within 20 days PSO (median, 13 days PSO) [128]. A correlation has been observed between the increase in serum blood concentrations of IgA and IgG anti-S proteins and decrease in the viral counts, as well as the time between the onset of symptoms and admission to the intensive care unit. Moreover, a significant relationship between the serum titers of anti-S IgA and IgG and the survival of patients in a critical condition has been demonstrated [130]. In addition to neutralization, Abs can result in antiviral protection through other mechanisms, like antibody-dependent cell cytotoxicity (ADCC) resulting from FcγRIIIa cross-linking in NK cells, antibody-dependent phagocytosis (ADCP) mediated by mononuclear and granulocyte phagocytes that bind to antibody-coated viruses through different Fc receptors, and complement activation by the classical route with the participation of IgM and IgG [131]. However, sometimes these same mechanisms can enhance the pathogenic condition, as previously described [107, 108]. In the case of COVID-19, there are studies that demonstrate that the plasma of convalescent patients contains Abs capable of mediating ADCC, phagocytosis, and complement activation [132].

## Insights from Ab therapeutic strategies against SARS-CoV-2 infection

### Immunoglobulins

**Fig. 2:**
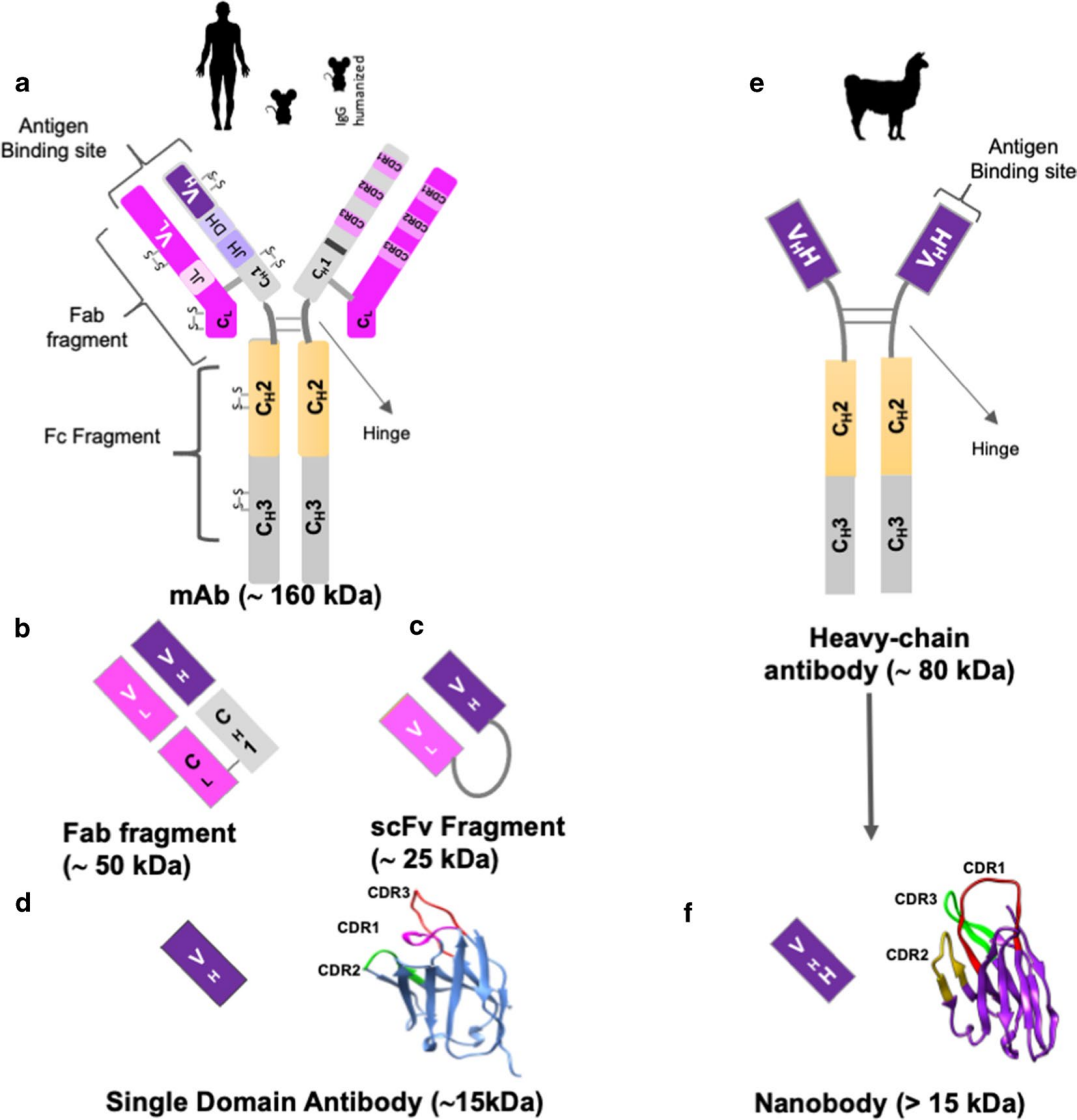
Diagram of antibodies and their respective fragments, from sources such as human, mouse, genetically humanized mouse, and alpaca. **a** mAb general view fragment antigen-binding region composed of two heavy and two light chains, disposed in Fab fragment and the fragment crystallizable (Fc) which consists of constant heavy chains (C_H_2 and C_H_3). The variable region formed by two arms which bind to antigen through complementary determining regions (CDRs). **b** Fab fragment is formed by the light chain (V_L_ and C_L_) and by the heavy chain’s variable (V_H_) region and a portion of its constant (C_H_1). **c** A single-chain variable fragment (scFv) comprises the fusion of the V_H_ and V_L_ of immunoglobulins, connected by a linker peptide. **d** Single domain antibody (nanobody) consists of a monomeric variable domain (V_H_) of a heavy-chain antibody of a common IgG. **e** Antibodies from Camelidae or heavy-chain antibodies, presenting a variable region of a heavy chain (V_H_H) and do not present light chains. **f** The V_H_H (Nanobody) derived from heavy-chain only antibodies have a longer CDR3 loop compared to V_H_-V_L_ domains in mAbs

Immunoglobulins are heterodimeric proteins comprising two identical 55-kDa heavy (H) chains and two identical 25-kDa light (L) chains linked by inter-chain disulfide bonds between conserved cysteine residues (Fig. 2) [133]. The evaluation of the immune response in patients infected with SARS-CoV-2 is of great importance to understand the production of Abs. One study showed that 13 of 14 patients presented IgG1 anti-S-RBD, and in two patients, the presence of IgG3 was observed, while IgG2 was not found [134]. Immunoglobulins from patients with non-severe and severe COVID-19 have affinity for the S protein or RBD that could block its interaction with hACE2, thereby preventing virus replication [16, 17, 32, 134–138]. An IgM response against the N protein, with a change in isotype to IgG after 15 days has been observed [13]. Although the titers of neutralizing Abs against SARS-CoV-2 in the human plasma decrease over time, these remain for at least three months until seroconversion [139].

A variety of anti-S or anti-RBD immunoglobulins generated from low somatic mutations are consistent with acute infection [13, 16, 17, 135, 140–144] due to low maturation of the affinity of Abs produced by B lymphocytes [17]. The neutralizing Abs have different epitopes, but many of them share the heavy-chain coding genes originating from similar germ lines of V-segments belonging to the VH3 family (VH3-23, VH3-30, VH3-53, or VH3-66), as well as VH169, VH2-70, and VH5-51 [16, 32, 135, 136, 138, 140, 142, 144, 145]. The light chains are preferably encoded by KV1-5, KV1-17, KV1-33, KV1-39, KV3-15, KV3-20, KV2-28, LV2-14, LV3-21, LV1-40, LV2-23, and LV6-57, among others [140, 142, 146]. Of note, these light chains predominantly pair with the long CDR H3 segment in the RBD region (15 amino acids or longer) [28, 135, 136, 138, 142, 145]. Whereas the other light chains pair with the short CDR H3 segment, which is 7–11 amino acids long [16, 32, 135, 136, 142, 145, 146].

### Convalescent plasma therapy: one of the ways to fight COVID-19

The implementation of the use of convalescent plasma (CP) has been a strategy to confer immunity or to treat individuals who acquire COVID-19. CP is collected from patients with neutralizing Abs after recovery and used to generate passive immunization [147–162].

**Fig. 3:**
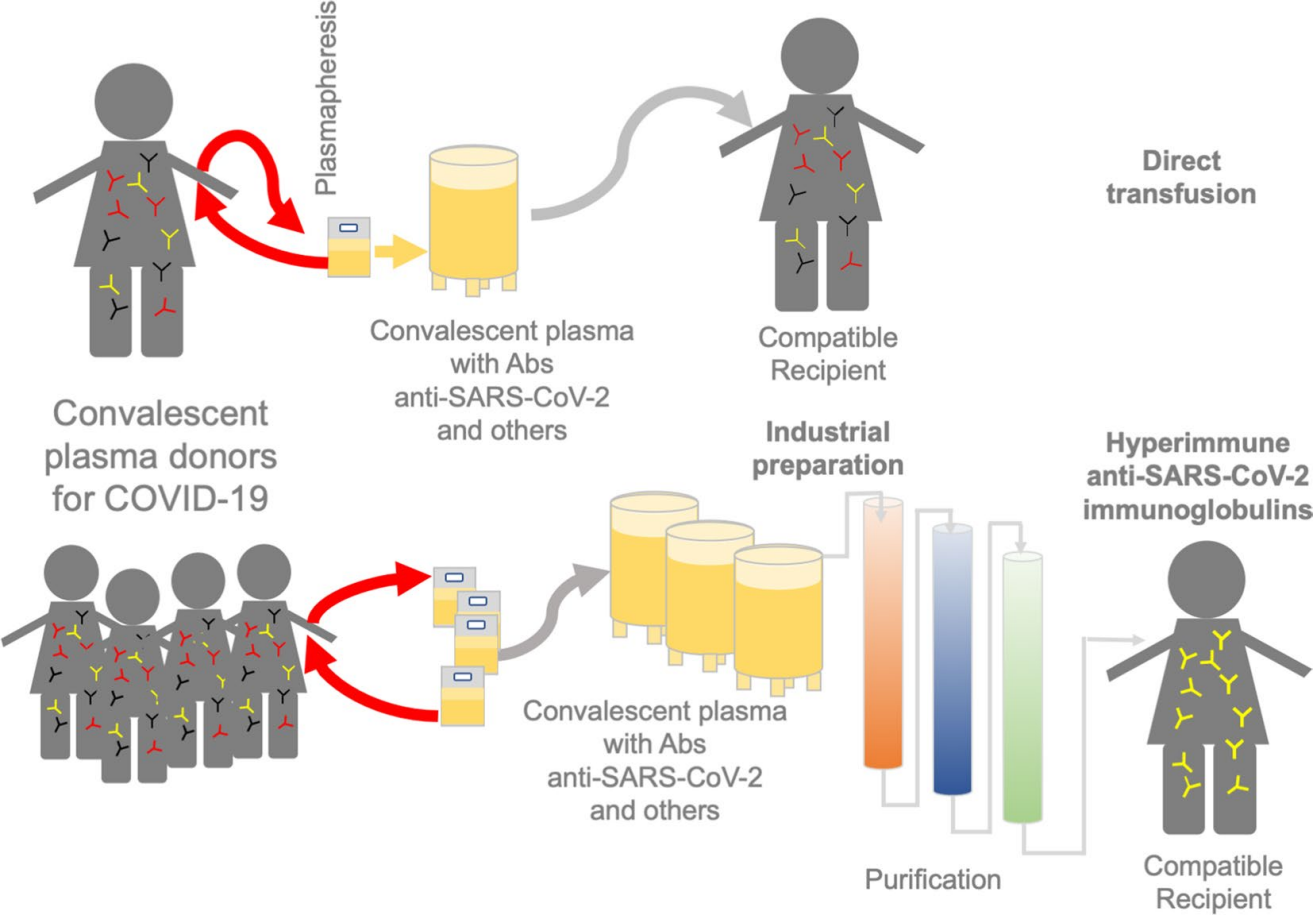
Methods of extraction and administration of Convalescent Plasma (CP). **a** a convalescent donor who has developed antibodies after recovering from the disease could donate plasma (usually through plasmapheresis) that includes antibodies against SARS-CoV-2 for direct transfusion and other antibodies (passive immunity) to patients with severe symptoms of the disease. **b** plasma from a group of donors could be used to identify and purify specific antibodies against SARS-CoV-2, eliminating other antibodies and proteins, making this method an alternative for passive immunization

The treatment involves collection of plasma from recovering patients, i.e., those who have faced an infectious disease and been cured successfully (known as convalescent patient), with the intention for it to be administered to recipient patients who have not yet developed an effective adaptive immune response (Fig. 3a) [163, 164]. The main objective of this alternative treatment is to reduce the viral load (viremia) in the recipient by the action of neutralizing Abs produced by the donor, which can occur between 10- and 14-days post infection [165, 166]. A crucial factor for the success of CP therapy is the selection of donors, since one of the main problems that have been identified is the diversity of virus variants found in the population, and the neutralizing Ab titer in different plasma samples [167]. Thus, it is necessary to ensure that the plasma contains an appropriate concentration of neutralizing Abs, determine the antibody titer, and use a neutralization test in vitro with the virus variants. However, the potential side effects of CP therapy must be considered, particularly the serum incompatibility in recipients [153, 154, 168–170]. The CP therapy used to treat SARS-CoV and MERS-CoV patients in the critical stage of infection has been shown to reduce the viral load and death rate [165, 171]. Based on the findings in the treatment of these diseases caused by other CoVs, CP from SARS-CoV-2-infected patients is administered as an experimental therapy in critically ill patients (Fig. 3a) (Additional file 1: Table S2). In addition, the use of polyclonal immunoglobulins and plasma derivatives isolated and purified from the blood of COVID-19 survivors has been discussed [172, 173] (Fig. 3b). CP administration in patients leads to an increment in IgG, IgM, and neutralizing Ab titers [147, 149, 150, 152], a decrease in short-term mortality in patients with severe respiratory failure [155] and hospital mortality, and the shortening of duration of admission in hospital for severely ill patients [156]. It is also suggested that CP treatment can be more efficient when it is administered to patients with no critical or life-threatening conditions [148, 160, 162]. CP therapy is accompanied by the supplementation of different medications, including antivirals, antibiotics, antifungals, corticosteroids, and anticoagulants [147–159], according to the patients needs, resulting in variations relative to the healthy subjects. Due to the simultaneous use of CP and other medications, it is inappropriate to determine the beneficial or adverse effects of CP therapy conclusively.

In a study administering CP to a group of 22 critically ill patients, the hospital mortality rate reduced by 55% compared with that observed in other studies (Additional file 1: Table S2), and the hospitalization length was reduced [156]. This is probably due to the high SARS-CoV-2 Abs titer of plasma administered more than once [156]. However, this study represents a compassionate bias when applying therapy to critically ill patients with little possibilities of success [162], unlike the treatment outcomes reported in other studies [148, 150, 160, 162], which showed no positive effects in such patients. Nonetheless, a precise design of controlled studies, randomized trials, and a high number of subjects are paving the way to further assess the benefits of CP therapy [152, 153, 156–158, 161]. It is important to mention that CP treatments depend on plasma collecting time. The neutralizing Abs from CP of COVID-19 patients are enriched between 31 and 35 days after the first symptoms presenting a higher neutralization titer [174], but decreases in titers over time (42 days after first symptoms) [175]. Hence, FDA recommends a minimum titer of neutralizing Abs of 1:160.

Importantly, the presence of different Abs with the ability to neutralize specific epitopes of the virus, as well as their biotechnological production and application as a life-saving therapeutic agent, still requires investigation.

### Neutralizing Abs against SARS-CoV-2

Due to their high Ag specificity and potency, Abs have been used for the treatment of different illnesses. Hence, identification and production of the best candidate Abs against the key epitopes of SARS-CoV-2 will be vital [12, 176]. Therefore, different strategies have been used to capture and obtain neutralizing Abs from patients with COVID-19 [16, 46, 177–180], such as combinatorial display libraries, humanized mice, single B cell cloning, memory B cell immortalization, and B cell culture, until the production of recombinant antibody fragments [46, 181–183] in different formats (Fig. 2). In this sense, Abs have shown a neutralizing effect in vitro and in vivo [18] (Table 1, Additional file 1: Table S3), although their safety and efficacy in vivo, as well as their contributions in ADCC, antibody-dependent genotoxicity, and even antibody-dependent risks are under evaluation, and there is scarce information on attempts of production on an industrial scale.

**Table 1:** Anti-SARS-CoV-2 antibodies whose structure and interaction with their respective epitopes have been described and based on them classified into the groups defined by Barnes et al. [194]

General view	Class [194]	Binding mode	Binding description	Sub-groups	mAb	K_*D*_ (nM)	IC_50_ (PSV-CoV-2) ng/mL	IC_50_ (AV-CoV-2) ng/mL	Status	PDB code	References
Overlap with hACE2-binding site	Class 1	hACE2-like binding mode	The binding to RBD in up conformation that mimics the interaction with hACE2		298 (multabody)	NR	28,000 (IgG) 0.11 (MB)	2200 (IgG) 5.7 (MB)	NR	7k9z	[196]
					910–30	0.162	66	180	NR	7ks9	[292]
					15,033	0.3 (IgG)	NR	489	NR	7klg	[293]
					15,033–7	0.039 (IgG)	NR	83	NR	7klh	[293]
					B38	70.1	NR	177	with H4 Protect hACE2 transgenic mice	7bz5	[146]
					BD-236	2.8	37	NR	NR	7chb	[140]
					BD-604	0.15	5	NR	NR	7ch4	[140]
					BD-629	0.14	4	NR	NR	7ch5	[140]
					C102	27	34	NR	NR	7k8m	[142, 194]
					C105	14	26.1	NR	Promising candidate	6xcn	[142, 194]
					C1A-B3	76.3 (RBD)	53	441,000	NR	7kfw	[180]
					C1A-B12	4.2 (RBD)	81.0	62	NR	7kfv	[180]
					C1A-C2	14.1 (RBD)	118	184,000	NR	7kfx	[180]
					C1A-F10	55.7 (RBD)	8	184,000	NR	7kfy	[180]
					CB6	2.49	41.0 (ND_50_)	36.0 (ND_50_)	CB6-LALA protects rhesus macaques	7c01	[204]
					CC12.1	5.92	19	22	Protect a hamster model	6xc2	[16]
					CC12.3	8.59	18	26	NR	6xc4	[16]
					COVA2-04	2.3	220	2.5	NR	7jmo	[136]
					CV07-250	0.056	NR	3.5	NR	6xkq	[17]
					CV30	3.63	NR	30	NR	6xe1	[144, 294]
					REGN10933^a^	0.041	0.042	0.037	Clinical trials	6xdg	[145, 201]
					S2E12	1.6 (RBD) 2.5 (S)	NR	5.29	NR	7k4n	[223]
					S2H14	75 (RBD) 90.1 (S)	900	NR	NR	7jx3	[178]
	Class 2	Overlap with hACE2-binding site	RBD binding mode in “up/down” conformation, that partially overlaps with hACE2 site, with angle of attack and positioning different from Class 1	Tertiary epitope	BD-368–2	0.82	1.2	15	Protect hACE2 transgenic mice	7chh	[32]
					C110	1.3	18.4	NR	NR	7k8v	[142, 194]
					COVA2-39	1.1 (RBD) 0.1(S)	36	54	NR	7jmp	[136]
					CV07-270	NR	NR	82.3	NR	6xkp	[17]
					DH1047	NR	90	124		7ld1	[294]
					H11-D4^b^	39.0	NR	18 nM	NR	6yz5	[30]
					H11-H4^b^	12.0	NR	6 nM	NR	6zhd	[13]
					LY-COV555	1.45 (FAB)	12,103	20–49	Clinical trials	7kmg	[200]
					MR17^c^	83.7 (RBD)	12,320	NR	NR	7c8w	[249]
					P2B-2F6	5.14	50	410	Preclinical	7bwj	[135]
					REGN10987^a^	0.042	40	42	Clinical trials	6xdg	[145, 201]
					S2H13	149(RBD) 119 (S)	500	NR	NR	7jv6	[178]
					SB23 ^3^	4.9 (Sb23 vs S) 0.225 (Sb23-Fc vs RBD)	NR	600.0 (Sb23) 7 (Sb23-Fc)	NR	7a29,7a25	[250]
					SR4^d^	14.5 (RBD)	5900	NR	NR	7c8v	[249]
				Quaternary epitope	2–4 Fab	NR	394	3	NR	6xey	[205]
					2–15	0.114	5	1	NR	7l5b	[206]
					BD23	NR	4800	NR	NR	7byr	[32, 189]
					C002	11.0	8.9	NR	NR	7k8t	[142]
					C104	19.0	23.3	NR	Promising candidate	7k8u	[142, 194]
					C119	10.0	9.12	NR	NR	7k8w	[142, 194]
					C121	0.5	6.73	1.64	Promising candidate	7k8x	[142, 194]
					C144	18.0	6.91	2.55	Promising candidate	7k90	[142, 194]
					mNB6^d^	0.56 (RBD) 0.45 (S)	6.3	12	NR	7kkl	[252]
					S2M11	66.0 (RBD) 68 (S)	NR	1.66	NR	7k43	[223]
No overlap with any residue of hACE2	Class 3	No cryptic epitopes	The epitope is exposed in RBD in up or down conformation		2–51	3.6	5	0.7	NR	7l2c	[207]
					C135	6.0 (RBD)	6.91	2.98	Promising candidate	7k8z	[142]
					DH1050.1	16 (Fab)	39	161		7lcn	[295]
					S309	0.3 (RBD) ~ 0.2 (S)	NR	79.0	Fc variant fast-tracked for clinical trials	6wpt	[4, 27]
	Class 4	Cryptic epitopes	Epitope exposed only in RBD up configuration		52^e^ (multabody)	NR	17 (IgG) 0.2 (MB)	6200 270.0 (MB)	NR	7k9z	[196]
					CR3022	6.3 (RBD)	NR	93 nM	Preclinical	6yor	[28–30]
					EY6A	2	NR	390	Promising candidate	6zdh	[13]
					H014^f^	0.09	3 nM	38 nM	Preclinical	7cak	[4]
					S304	4.58 (RBD)	NR	> 5000	NR	7jw0	[27]
					S2A4	7.5 (RBD) 10 (S)	3500	NR	NR	7jvc	[178]

*AV* authentic virus**,** *IC*_*50*_ half-maximal inhibitory concentration**,** *K*_*D*_ dissociation constant**,** *MB* multibody**,** *ND*_*50*_ 50% neutralization dose**,** *NR* not reported**,** *PSV* pseudovirus

^a^Human/IV mice

^b^Llama source

^c^Sybody

^d^Nanobody

^e^New fusion antibody protein

^f^Obtained by phage display

#### Targets and classification of Abs against SARS-CoV-2

**Fig. 4:**
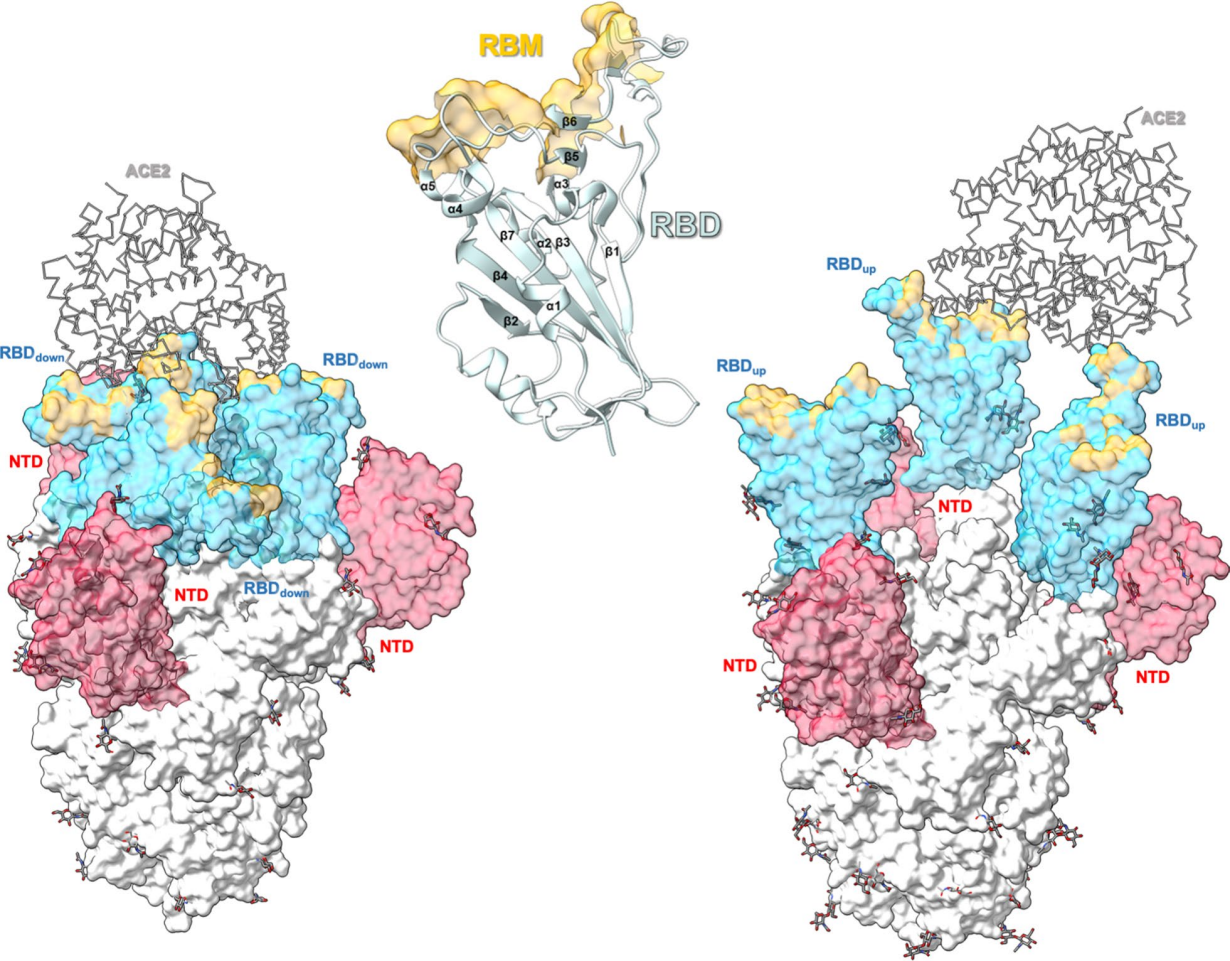
Schematic representation of the homotrimeric S structure. The S protein conformations with all “down” (left) and all “up” (right) RBDs were generated with PDB files 7k90 and 7k4n, respectively. The RBM of RBD (center) is highlighted as an orange surface

A variety of Abs targeting different epitopes of SARS-CoV-2 have been described, principally those against the β-coronavirus envelope (Table 1, Additional file 1: Table S3), conformed more externally by S protein [19, 177]. The S protein is a highly glycosylated homotrimeric protein of ~ 180–200 kDa (Fig. 4). As an inactive precursor, each S protomer (1273 residues) comprises two functional regions that become active after cleavage by the human protease TMPRSS2 [9–11]. The S1 subunit (14–685 residues) triggers the invasion process by mediating virus binding to the N-terminal domain (NTD) of hACE2, while the S2 subunit (686–1273 residues) drives the fusion of the viral and cellular membranes (Fig. 4), similar to that noted for other CoVs [9–11, 184].

The S1 subunit is composed of two domains, the NTD (14–305 residues) bearing a galectin-like motif and an RBD (319–541 residues) having a core comprising five-stranded antiparallel β-sheets (β1–4 and β7) connected to helices (α1–α3) and loops. The receptor-binding motif (RBM) within the RBD interacts with hACE2 at the 446–505 residue segment (Fig. 4) [12, 185–187]. The three RBDs undergo a hinge-like conformational equilibrium change from a “down” or closed pre-fusion state to an “up” or open fusion-prone state [14, 36, 188–190]. In the pre-fusion conformation, each RBD contacts extensively with the other RBDs and its own intracatenary NTD partially burying the RBM. Therefore, hACE2 binding is only possible in the active S protein conformation, with RBDs in the “up” state [184, 188, 191–193].

**Fig. 5:**
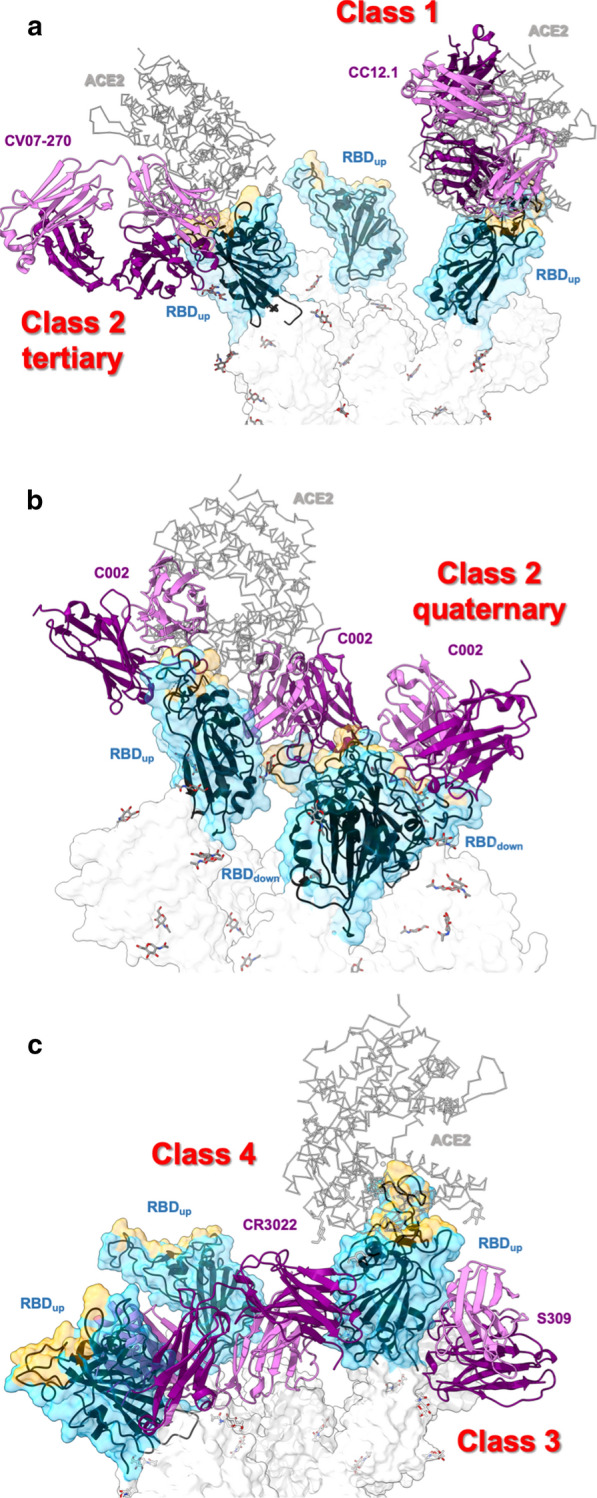
Classes of antibodies according to the binding pose. Coordinates for antibodies CC12.1 (Class1), CV07-270 (Class 2 tertiary), C002 (Class 2 quaternary), S309 (Class 3), and CR3022 (Class 4) were taken from PDB files 6xc2, 6xkp, 7k8t, 6wpt, and 6yro, respectively

The neutralizing anti-SARS-CoV-2 Abs isolated from CP often recognize the RBD, particularly the RBM, and thus, interfere with the virus-hACE2 interaction and prevent viral particle entry into the target cell [12, 17, 32, 135, 194]. Approximately 78% and 70% of COVID-19 CPs have been found to present anti-RBD and anti-S IgG, with those in hospitalized individuals having high neutralizing activities [142]. This interaction bias is due to the RBM being an immunodominant region [178] and the RBD/spike protein-based strategies used to isolate many of these Abs. To date, several dozen structures of different Abs bound to the isolated RBD or S protein have been resolved experimentally (Table 1). According to their mode of binding to the viral protein, they have been grouped into four classes [194] (Fig. 5, Table 1). Class 1 comprises the largest group of Abs. These are characterized by a short CDRH3 loop and a binding pose that resembles the angle of interaction with hACE2, largely overlapping the RBM (Fig. 5a). Thus, these Abs can only bind to the “up”-state RBDs. Class 2 comprises Abs that partially overlap with the hACE2 footprint and can recognize both “up”- and “down”-state RBDs (Fig. 5a, b). As with Class 1, the binding poses of Class 2 Abs show that the neutralization effect is due to direct competition with hACE2, consistent with the competitive binding assay findings. The ability of Class 2 Abs to bind RBDs in both the conformations results from their different angles of interaction with hACE2, avoiding any steric hindrance with the other RBDs even in the “down” conformation. Class 2 includes Abs with a long CDRH3 loop. Interestingly, some Class 2 Abs simultaneously bind two RBDs. Different interaction patterns have been observed for these “quaternary Abs.” For example, the long CDRH3 loop of the mAb C144 interacts with two “down”-state RBDs. In this way, three C144 Abs lock the S protein in the pre-fusion conformation [194]. Additionally, of the three C002 Abs that bind to the S protein, one links the other two “down”-state RBDs, the second binds one “down”-state and one “up”-state RBD, while the third binds only the “up”-state RBD (Fig. 5b) [194].

Classes 3 and 4 include Abs that bind outside the RBM. Some of these Abs compete directly with hACE2 because of the relative proximity of their epitope to the RBM. In the case of the mAb EY6A, steric hindrance occurs because of collision with hACE2 glycans [13]. Other mAbs do not interfere with the binding of hACE2 to the same RBD to which it is bound, but rather with that to a neighboring RBD. Class 3 Abs recognize a solvent-exposed protein/glycan epitope in both “up”- and “down”-state RBDs (Fig. 5c). This epitope is highly conserved in *Sarbecovirus* clades 1, 2, and 3 [27, 195], making it more difficult for the viruses to develop escape mutations. Class 4 Abs bind cryptic epitopes that become accessible only in “up”-state RBDs (Fig. 5c). One of these epitopes is buried by the contact between “down”-state RBDs. Therefore, Abs that recognize this epitope tend to affect the conformation and/or binding capacity of adjacent RBDs. An extreme case is represented by the mAb CR3022, which promotes destruction of the pre-fusion S protein trimer by perturbing the folding of both NTDs and RBDs [30, 33, 36]. mAb 52 recognizes a different cryptic epitope that is buried by the NTD in the pre-fusion conformation [196]. Although most Abs recognize epitopes consisting of only peptide moieties, some of them bind to protein/glycan moieties, sometimes with very high neutralizing potency [197, 198].

#### Abs with cross-neutralizing activity against SARS-CoV-2

The battery of neutralizing Abs described so far has been the result of intensified research using samples from various sources (Additional file 1: Table S3, S4). Since the RBDs of SARS-CoV and SARS-CoV-2 are ~ 75% identical in their primary sequence, only a relatively small number of Abs have shown cross-reactivity with these two Ags [12, 23]. The first set of anti-SARS-CoV-2 Abs was obtained from the blood of patients with anti-SARS-CoV Abs [13, 27]. In the initial studies, cross-neutralization was scarcely noted [15, 27, 29, 197, 198]. However, some anti-SARS-CoV Abs have shown cross-neutralizing activity against SARS-CoV-2 [12, 27]. For instance, the neutralizing Ab S309 obtained from B cells from a patient infected with SARS-CoV [27] and CR3022 IgG and Fab isolated from SARS-CoV CP present cross-reactivity with the SARS-CoV-2 RBD [28, 29, 36] (Additional file 1: Table S3). Another study has reported a 47D11 SARS-CoV-neutralizing Ab that also neutralized SARS-CoV-2 [12, 33]. Data suggest a cross-neutralizing epitope shared between both the CoVs, which is directly related to some of the epitopes conserved in the RBD [194]. Despite this, the Fab 2G12 developed as anti-HIV-1 presents cross reativity towards the S2 domain via glycan recognition, being of interest for the design of new therapies [199].

#### Useful Abs with therapeutic or profilactic efficacy

At least 80 mAbs have been shown to block the interaction of the RBD with the hACE2 receptor in vitro, with a neutralizing effect against a pseudovirus or the authentic SARS-CoV-2 (Additional file 1: Table S3), and around 30 mAbs are under clinical trials (Additional file 1: Table S4) [52, 53]. Among these, bamlanivimab (LY-CoV555) developed by Eli Lilly and Company (Indianapolis, IN, USA) has been granted emergency use authorization by the FDA [52, 200]. Similarly, two potent Abs, REGN10933/casirivimab and REGN10987/imdevimab (Regeneron, Tarrytown, NY, USA), developed and recovered from VelocImmune® (Regeneron), a genetically modified mouse with a human immune system, form part of the REGN-COV2 treatment, which is authorized by the FDA for emergency use [48, 52, 53, 145]. Each Ab recognizes the RBD at distinct sites, increasing the protection against and avoiding the escape of the virus by most of mutations [145, 201]. The REGN-COV2 cocktail or LY-CoV555 have demonstrated that decrease viral load and reduce the risk of progression to severe COVID-19 and hospitalization, each one with a particular dose [52, 202]. Although a recent study of LY-CoV555 did not demonstrate efficacy coupled with supportive care (remdesivir and, when indicated, supplemental oxygen and glucocorticoids) in hospitalized patients without end-organ failure, using a high dose (7000 mg per patient) [203].

Furthermore, several mAbs have demonstrated effectiveness in preclinical studies, and are currently under clinical trials of different phases (Additional file 1: Table S4). Some of these include sotrovimab, AZD7442, regdanvimab, DXP-593, DXP-604, etesevimab, STI-1499/COVI-SHIELD, CT-P59, TY027, SCTA01, MW33, HFB30132A, BRII-196, BRII-198, ABBV-47D11, ABBV-2B04, COVI-GUARD (STI-1499), COVI-AMG (STI-2020), ADM03820, DZIF-10c, AD-20, JMB2002, LY-CovMab, C-144-LS, C-135-LS, COR-101, JS016, and HLX70 (Additional file 1: Table S4). The human Ab VIR-7831 (or GSK4182136), developed by Vir Biotechnology Inc. (San Francisco, CA, USA) and GlaxoSmithKline (Brentford, UK), presents an epitope similar to that of S309 (Table 1, Fig. 5) and is presently under phase 3 evaluation [52, 53]. CT-P59 (Celtrium) and ADG20 (Adagio Therapeutics) is under phase 2/3 clinical trial. The Abs AZD8895 and AZD1061, developed by Vanderbilt University and licensed by AstraZeneca (Cambridge, UK), TY027 (Tychan Pte., Ltd., Singapore), are in phase 3 clinical trial. In addition to etesevimab (LY-CoV016 or JS016) developed by Eli Lilly and Company, another related Ab CB6LALA is under phase 3 clinical trial [204]. Bamlanivimab (700 mg/dose) in combination with etesevimab (1400 mg/dose) won FDA authorization for emergency use. Other Abs, such as DXP-593 related to BD-368–2 and SCTA01, are close to completing phase 2 or phase 2/3 clinical trials. Whereas Abs such as STI-1499/COVI-SHIELD (Sorrento Therapeutics Inc., San Diego, CA, USA), BRII-96 (Brii Biosciences, Durham, NC, USA), BRII-98 (Brii Biosciencies), ABBV-47D11 (AbbVie, North Chicago, IL, USA), COVI-GUARD (STI-1499; Sorrento Therapeutics Inc.), COVI-AMG (STI-2020; Sorrento Therapeutics Inc.), MW33 (Mabwell Bioscience Co., Ltd., Shanghai), HFB30132A (HiFiBiO Therapeutics, Cambridge, MA, USA), and HLX70 (Hengenix Biotech Inc., Fremont, CA, USA) are under phase 1 clinical trials (Additional file 1: Table S4). Among the Abs in clinical trials, the characterization of Abs such as CB6-LALA and BD-368-2 [32, 204] has been fundamental. CB6-LALA is a neutralizing mAb isolated from B cells from the CP of patients with COVID-19, and it blocks the binding between the SARS-CoV-2 RBD and hACE2 through steric hindrance and competition for the interface amino acid interaction, without inducing conformational changes in the RBD. It has been proposed as a potential therapeutic agent against SARS-CoV-2 in rhesus macaques because it reduces the viral titer and infection [204]. This Ab has been modified via leucine-to-alanine mutations at residues 234 and 235 (LALA mutation) in the Fc region to diminish the possibility of Fc-mediated acute lung injury [204]. In addition, BD-368–2 effectively neutralizes the pseudovirus of SARS-CoV-2 and authentic SARS-CoV-2 (IC_50_ of 1.2 ng/mL and 15 ng/mL, respectively) [32] (Table 2). BD-368-2 binds the RBD in the “down” conformation, localizing between the NTD and RBD and adjacent to an RBD in the “up” conformation. Moreover, BD-368-2 can bind RBDs in the “up” and “down” conformations, to reach complete occupancy of the S protein trimer [140] (Table 1, Table 2). BD-368-2 can interact with the RBD in combination with CR3022 and S309 [27, 29]. Furthermore, BD-368-2 shows prophylactic and therapeutic efficacy (Additional file 1: Table S3) in hACE2 transgenic mice [32].

**Table 2:** Antibodies with a potent neutralizing effect against pseudovirus or authentic virus SARS-CoV-2 infection

Name/class	Source	K_*D*_ (nM)	IC_50_ μg/mL	Target	Observations	References
CV07-250/C1	B cells from C-CoV-2	0.056	0.0035 (AV-CoV-2)	RBD	Reduced hACE2 binding and showed no binding to murine tissue	[17]
BD-604/C1	B cells from C-CoV-2	0.15	0.005 (PSV-CoV-2)	RBD up	BD-604 binds to RBD ~ 19-fold higher than BD-236 and is more potent against the SARS-CoV-2 pseudovirus, compared to BD-236	[140]
BD-629/C1	B cells from C-CoV-2	0.006	0.004 (PSV-CoV-2)	RBD up	Genes coding for BD-629 are different compared to BD-604. However, its affinity and neutralization against the SARS-CoV-2 pseudovirus are similar	[140]
CV07-209/C1	B cells from C-CoV-2	0.056	0.003 (AV-CoV-2)	RBD	Prophylactic and therapeutic efficacy in golden Syrian hamsters. Therapeutic mAb reduced signs of COVID-19, although 1/3 animals presented mild bronchopulmonary, pneumonia and endothelialitis	[17]
COVA1-18/C1	B cells from C-CoV-2	0.03 (S) 0.9 (RBD)	0.008 (PSV-CoV-2) 0.007 (AV-CoV-2)	RBD	A strong competition with hACE2 was observed, suggesting blocking hACE2 is it mechanism of neutralization	[136]
CC6.29/C1	B cells from C-CoV-2	1.2	0.002 (PSV-CoV-2) 0.0071 (AV-CoV-2)	RBD-A	mAb exhibited a potent neutralization	[16]
COV2-2196/C1	B cells from C-CoV-2	–	0.0007 (PSV-CoV-2) 0.015 (AV-CoV-2)	S2P_ecto_ open	A strong competition with hACE2. Prophylactic efficacy in rhesus macaques (50 mg/Kg) and mice (200 µg per mouse) reducing lung disease. Therapeutic efficacy in mice (20 mg kg^−1^)	[208]
BD-368–2/C2	B cells from C-CoV-2	0.82	0.0012 (PSV-CoV-2) 0.015 (AV-CoV-2)	RBD “up/down”	Changes the S trimer conformation contributing to its neutralizing activity. Prophylactic efficacy: IP 20 mg/kg mAb 24 h before infection. Therapeutic efficacy: IP 20 mg/kg of mAb 2 h after infection into hACE2 transgenic mice	[32]
COV2-2130/C2	B cells from C-CoV-2	–	0.0016 (PSV-CoV-2) 0.107 (AV-CoV-2)	S2P_ecto_ closed	Blocked the binding of SARS-CoV-2 to hACE2. Prophylactic efficacy in rhesus macaques (50 mg/Kg) and mice (200 µg per mouse) developing less lung disease. Therapeutic (20 mg kg^−1^) efficacy in mice	[208]
C12-04/C2	B cells from C-CoV-2	2.3 (S) 11.2 (RBD)	0.220 (PSV-CoV-2) 0.002 (AV-CoV-2)	RBD “up”/“down”	Potent neutralizing mAB, suggest the blocks the engagement of hACE2 as a main mechanism of neutralization	[136]
C119/C2	PMBC´s from C-CoV-2	10.0 (RBD)	0.009 (PSV-CoV-2)	RBD “up”/“down”	It was proposed a quaternary interaction with RBD in down conformation adjacent to an “up” RBD, as well could interacts between two adjacent down RBD domains. Showed a binding pose similar to REGN10987′s	[142, 194]
C121/C2	PMBC´s from C-CoV-2	0.5 (RBD)	0.0067 (PSV-CoV-2) 0.00164 (AV-CoV-2)	RBD “up”/“down”	Quaternary binding with RBD in down adjacent to an “up” RBD was proposed, and could interacts between two adjacent down RBD, with a binding pose similar to REGN10987′s	[142, 194]
C144/C2	PMBC´s from C-CoV-2	18.0 (RBD)	0.0069 (PSV-CoV-2) 0.0025 (AV-CoV-2)	RBD “up”/“down”	Quaternary binding, in the “down” RBD conformation. different from C002, C121, C119, C104	[142, 194]
COVA2-15/C2	B cells from C-CoV-2	0.6 (S) 3.1 (RBD)	0.008 (PSV-CoV-2) 0.009 (AV-CoV-2)	RBD “up”/“down”	A strong competition with hACE2 binding, binding RBD in "up" and "down" conformations, while its epitope is partially overlapped with the hACE2-binding site	[136]
2–15/C2	B cells from C-CoV-2	0.056	0.005 (PSV-CoV-2) 0.0007 (AV-Cov-2)	RBD “up”/“down”	Exhibited high potency in neutralizing in vitro, in a protection experiments using golden Syrian hamster reduced the infectious virus titres by 4 logs (1.5 mg/kg)	[205, 206]
C002/C2	PBMC from C-CoV-2	11 (RBD)	0.009 (PSV-CoV-2)	RBD “up”/“down”	Quaternary binding to “up/down” RBDs like C121, but different to C144. Interaction with RBD in down conformation adjacent to an “up” RBD, probably interacts between two adjacent "down" RBD domains	[142, 194]
C135/C3	PMBC´s from C-CoV-2	6.0 (RBD)	0.016 (PSV-CoV-2) 0.0029 (AV-CoV-2)	RBD “up”/“down”	Three C135 Fabs bound with 2 “down” and 1 “up” RBDs (interaction weakly resolved), recognizing the glycosylated epitope N343RBD, interacting with R346 and N440, without steric hindrance between hACE2 / RBD	[142, 194]
2–51/C3	B cells from C-CoV-2	3.6	0.005 (PSV-CoV-2) 0.0007 (AV-Cov-2)	NTD	Potent neutralizing antibody against PSV-CoV-2 and AV-Cov-2 in vitro	[207]
H014/C4	phage display antibody library	0.09	3 nM (PSV-CoV-2) 38 nM (AV-CoV-2)	RBD up class 4	hACE2-humanized mice injected IP 50 mg per kilogram either 4 h after (one dose, therapeutic) or 12 h before and 4 h after (two doses, prophylactic plus therapeutic) with SARS-CoV-2 infection. No lesions of alveolar epithelial cells	[4]
5–24/WO	B cells from C-CoV-2		0.013 (PSV-CoV-2) 0.008 (AV-CoV-2)	NTD	nAb with high potency against AV-Cov-2 in vitro	[205]
1–57/WO	B cells from C-CoV-2	0.056	0.009 (PSV-CoV-2) 0.008 (AV-CoV-2)	RBD	mAb exhibited high potency in neutralizing AV-Cov-2 in vitro	[205]
2–7/WO	B cells from C-CoV-2	0.056	0.010 (PSV-CoV-2) 0.003 (AV-CoV-2)	RBD	mAb exhibited high potency in neutralizing AV-Cov-2 in vitro	[205]

*SARS-CoV* Severe acute respiratory syndrome–coronavirus, *SARS-CoV-2* respiratory syndrome–coronavirus 2, *RBD* Receptor binding domain,* PBMCs* Fresh peripheral blood mononuclear cells, *IP* Intraperitoneally, *PSV* Pseudovirus, *AV-CoV-2* authentic virus SARS-CoV-2, *AV-CoV* authentic virus SARS-CoV, *SdAb* single-domain antibodies, *CPE* Cytopathic effect, *N-t* amino-terminus, *C-CoV-2* Convalescent SARS-CoV-2, *NTD* N-terminal domain ((residue 1–290), *S2P*_*ecto*_ S ectodomain trimer (S_ecto_), *C1* Class1, *C2* Class 2, *C3* Class 3, *C4* Class 4, *WO*  those without structure analysis

#### Potent neutralizers mAbs against SARS-CoV-2

At least 21 Abs published present a potent neutralizing effect against the pseudovirus or authentic SARS-CoV-2 infection, with an IC_50_ lower than 0.01 µg/mL (Table 2). Among these, CV07-250, BD-604, BD-629, COVA1-18, CC6.29, COV2-2196 are Class 1 Abs (Table 2). Class 2 Abs such as BD-368-2, COV2-2130, COVA2-04, C119, C121, C144, COVA2-15, 2–15 and C002 are the most potent (Table 2). The Class 3 Ab C135 and 2–51, and Class 4 Ab H014 present an elevated neutralizing effect (Table 2). The mAb 5–24 that binds the NTD, and Abs CV07-209, 2–15, 1–57, and 2–7 that interact with the RBD, also present potent neutralizing activities (Table 2). The diversity in amongst the potent neutralizing Abs is crucial, considering their probable combinational use to achieve rational therapeutic effectiveness, as well as their usefulness in reducing or preventing evasion by the present or future virus variants.

The Abs CV07-250 and CV07-209 have been isolated from the B cells of patients with COVID-19, with CV07-209 being the most potent mAb that neutralizes authentic SARS-CoV-2 (IC_50_: 3.1 ng/mL) and CV07-250 presenting close enough activity (IC_50_: 3.5 ng/mL). The crystal structure of CV07-250 in complex with the SARS-CoV-2 RBD has been resolved at 2.55 Å. Prophylactic and therapeutic evaluations have shown CV07-209 to protect hamsters from SARS-CoV-2 infection [17]. CV07-250 (Class 1 Ab) binds the RBD, at a site overlapping with the hACE2-binding site, via an unusual light chain-dominated interaction. On the contrary, BD-604 and BD-629 (Class 1 Abs) also show a potent neutralizing effect against the SARS-CoV-2 pseudovirus with an IC_50_ of 5 ng/mL and 4 ng/mL, respectively. Both Abs interact with the RBD, with the binding of BD-629 dominated by the heavy chain in comparison with that of BD-604 [140]. The crystal structures of BD-604 and BD-629 resolved at 3.2 Å and 2.7 Å, respectively, show that they bind the RBD in a manner similar to that of other Class 1 Abs [140], such as C105 [142, 194] and CB6 [204] (Table 2).

A family of Abs obtained from the B cells of patients with COVID-19, including 2–15, 1–57, 2–7, and 5–24, have been found to have potent neutralizing activities against authentic SARS-CoV-2 in vitro with an IC_50_ of 0.7 ng/mL, 8 ng/mL, 3 ng/mL, and 8 ng/mL, respectively [205, 206]. The 1–57, and 2–7 Abs belong to Class 1. The 5–24 and 2–51 Ab binds the NTD and exerts a powerful neutralizing effect [205, 207]. The 2–15 Ab (Class 2) has been evaluated in vivo in protection experiments using golden Syrian hamster as a model of SARS-CoV-2 infection. Virus challenge showed a reduction in infectious viral particle titers with 1.5 mg/kg of 2–15 [205].

The Abs COVA1-18, COVA2-04, and COVA2-15 are also obtained from B cells of patients with COVID-19, and these present strong competition for hACE2 with an IC_50_ against authentic SARS-CoV-2 of 7.0, 2.0, and 9.0 ng/mL, respectively [136]. COVA1-18 appears to be a Class 1 Ab, and the cryogenic electron microscopy (cryo-EM) reconstructions reveal that COVA2-15 is able to bind RBDs in the “up” and “down” conformations and therefore, belongs to Class 2 [136]. The epitope and approach of binding to the RBD used by COVA2-04 is similar to that by CR3022, and hence, is categorized to Class 4 [28–30, 136] (Table 2).

The mAbs COV2-2196 and COV2-2130, which bind near the hACE2-binding site, exhibit powerful neutralizing activities. These mAbs present, in pseudovirus neutralization assays, an IC_50_ of 0.07 ng/mL and 1.6 ng/mL, respectively, although they are less sensitive for neutralization of the authentic virus (IC_50_: 15 ng/mL and 107 ng/mL, respectively). Both mAbs recognize the RBD in the “up” configuration, although they do not compete with the virus for binding with hACE2. Furthermore, COV2-2130 presents different competitive binding sites and is able to interact with an RBD in the “down” state, indicating that it could recognize the RBD in the “up” or “down” conformations by probably binding to three distinct sites on the S protein trimer [208]. Due to their differences in binding, COV2-2196 and COV2-2130 has been tested for prophylactic efficacy using a mouse-adapted SARS-CoV-2 model [208]. A cocktail of COV2-2196 (16 ng/mL) and COV2-2130 (63 ng/mL) presents a synergistic effect on virus neutralization in vitro compared with the effect observed by using 250 ng/mL of the Abs individually [208].

The Abs C002, C119, C121, C135, and C144 obtained from peripheral blood mononuclear cell of patients with COVID-19, interact with the RBD using different binding modes and present a strong pseudovirus neutralization effect (IC_50_: 9.0 ng/mL, 9.0 ng/mL, 6.7 ng/mL, 16.0 ng/mL, and 6.9 ng/mL, respectively). Moreover, the Abs C121, C135, and 144 also neutralize authentic SARS-CoV-2 (IC_50_: 1.6 ng/mL, 2.9 ng/mL, and 2.5 ng/mL, respectively) [142]. C002, C119, and C121 bind both the “up”- and “down”-state RBDs, where the Fab-S structures suggest a quaternary epitope, including the neighboring RBDs, to also support bivalent interactions with two “down”-state RBDs. Additionally, C002 seems to be in contact with glycans in the RBD [142]. C135 and C144 bind the same “up” RBD conformation. In addition, C135 binds a “down”-state RBD regardless of the conformation of the neighboring RBDs. The conformational changes in the RBD allow the configuration of a quaternary epitope, which is formed by neighboring “down”-state RBDs that are recognized by C144 through its long CDRH3 loop. This unique interaction locks the S protein domains in a pre-fusion conformation, thereby avoiding the S protein-open conformation, in which it engages with hACE2 [142, 194] (Table 2).

The humanized mAb H014 has been obtained from an Ab library constructed by phage display from immunized mice with recombinant RBD from SARS-CoV [4]. H014 neutralizes the SARS-CoV-2 pseudovirus infection (IC_50_: 3 nM) and authentic SARS-CoV-2 infection (IC_50_: 38 nM). Cryo-EM characterization of H014 Fab in complex with the SARS-CoV-2 S protein trimer suggests a novel conformational RBD epitope accessible in an “open” conformation, where the mAb interacts with the S protein and blocks the hACE2 engagement by steric hindrance and the associated protein–protein interactions, different to the RBM interaction [4]. Interestingly, H014 is capable of neutralizing in vivo in a hACE2 mouse model with a prophylactic dose [4] (Table 2). This Ab is categorized in Class 4, together with CR3022, EY6A, S304, and S2A4 [4, 13, 27, 28, 178]. In general, Abs grouped in Class 4 need the highest concentration to reach the neutralization effect compared with Abs from the other classes.

#### Mutants could reduce Abs neutralization

RBD mutations have been related with the reduction of the sensitivity or confer resistance to neutralizing Abs. For instance, mutations N439K, L452R, A475V, V483A, E484K, G485D, F486A, F490L, and Y508H weaken the binding of mAbs, such as 157, 247, CB6, P2C-1F11, B3SCA1, X593, 261–262, H4, P2B-2F6, H014, and H00S022 [4, 19, 30, 135]. The V483A variant, with a mutation frequency greater than 0.1%, elicits a loss of activity of mAbs, such as P2B-2F6 and X593 [19, 135]. While V_H_-Fc ab8 loss neutralizing activity against F486A mutant [209]. As well as REGN10933 showed a reduction of the sensitivity against E484K and G485D [145]. Similarly, RBD variants such as Q414E, N439K, G446V, K458N, I472V, A475V, T478I, V483I, and F490L cause viral resistance to CP [19]. In addition, variants such as N439K and Y508H are increasing in circulation [19]. Moreover, variants in the RBD or S_1_ subunit, which allow the viral particle to increase its transmissibility, pathogenicity, infectivity, and resistance, will continue to occur. D614G is a frequent mutation that does not occur in the RBD [48]. Although it has been related to SARS-CoV-2 infectivity and worsened COVID-19 symptoms, its participation in virus resistance has scarcely been demonstrated [48]. In contrast, Abs such as 2H2, 3C1, CC6, CC12, and CC25 neutralize the D614G variant [16, 48, 210, 211]. As well as STE90-C11 recognized with elevated affinity RBD mutations like V367F, N439K, G476S, V483A, E484K, G485R, F486V [212]. These data indicate the importance of using different Abs to achieve therapeutic effectiveness.

#### Cocktails of Abs

Based on distinct epitopes conserved in the S protein domain, a cocktail of neutralizing Abs has been used to mitigate the risk of COVID-19. Such cocktails can significantly enhance the neutralizing abilities [48, 143, 213, 214]. For example, to complement the neutralizing effect of H014 it was combined with the non-competitive antibody P17 obtained from a library of naive human antibodies. P17 has high affinity for RBD, and a potent neutralizing activity with pseudovirus (IC_50_: 0.165 nM) and highest IC_50_ against the authentic virus than H014. According to the authors, the cocktail of P17 and H014 improves (two to ten-fold) the protective effect against SARS-CoV-2 in mouse model [213].

Other combinations such as B38+H4, REGN10933+REGN10987, AZD8895+AZD1061, 414-1+555-63+553-15, COV2-2196+COV2-2130, and CR3022+CR3014 have been evaluated [26, 48, 214]. In fact, the addition of 553-15 to 414-1+555-63 or the combination of COV2-2196 with COV2-2130 has been shown to provide a synergistic neutralization effect [208, 214]. Furthermore, cocktails may have therapeutic potential in a possible SARS-CoV-2 reinfection, which has not been noted for other therapies [34, 215].

#### Abs Fc-mediated effector functions

The Fc-mediated effector functions, such as ADCC or ADCP, can contribute to virus clearance independent of the mAb neutralization effect [216, 217]. Briefly, infected cells may expose the Ags on the pathogen surface that can also be recognized by IgG, which through its Fc region binds the Fcγ receptors (FcγRs) and can attract other cells. In addition, IgGs bind C1q, drifting away from the complement-dependent cytotoxicity (CDC) pathway, which involves the IgG-bound Ag and recognition of the C1 complex [218]. Cytotoxic Abs, such as alemtuzumab, dinutuximab, and ofatumumab, present their main mechanism of action through ADCC and CDC. In fact, the Fc region of an Ab determines its serum half-life and effector functions, which are associated with the N-glycan structure [219, 220]. In particular, the absence of the fucose residue at the core increases the ADCC [221, 222].

Pinto et al. [27] demonstrated that S309 mediates ADCC in SARS-CoV-2 S protein-transfected cells, along with the strongest ADCP response by monocytes, among the immune cells, via FcγRIIIa and FcγRIIa engagement and affinity for an FcγRIIIa variant (V158). It also activates the CDC pathway [178]. S306 activates ADCC and ADCP with intensity lesser than that of S309. S2M11 promotes FcγRIIIa-dependent ADCC in a dose-dependent manner and does not promote FcγRIIa-mediated ADCC, with a high affinity towards the V158 variant comparable to that of S309 [27]. Moreover, S2M11 also exerts ADCP. S2E12 triggers FcγRIIa but not FcγRIIIa signaling, unlike S2M11 and S309. Furthermore, a combination of S2M11 with S2E12 or S309 activates effector functions [223]. S2H13 promotes ADCC through FcγRIIIa (V158) activation, but presents a weak activation of FcγRIIa. Additionally, S2H13 is effective in killing Chinese hamster ovary (CHO) cells stably transfected with SARS-CoV-2 S protein via CDC. A superior ADCC response by REGN10987 compared with REGN1089, REGN10933, and REGN10934 has been observed, but all Abs have been shown to induce ADCP [145]. These findings highlight the differences in Abs and their relationship with FcγRIIIa and FcγRIIa receptors, or C1q, which can be decisive in displaying their protective mechanisms [27, 178, 223].

### Hybridoma Abs

The recent discovery of potent antibodies has been driven using different technologies [46] instead of the traditional hybridoma production, although some Abs were also obtained by this strategy. Hybridomas were introduced in 1975 by Köhler and Milstein [224], and these are cloned cell lines produced by the fusion of a B lymphocyte of interest and an immortalized myeloma cell, which are capable of secreting large quantities of pure Abs [225]. Although hybridoma development represents a labor-intensive and time-consuming process [226], research related to hybridomas has continued over time. Accordingly, COVID-19 research has not excluded hybridomas, as there are multiple studies using this strategy for treating SARS-CoV-2 infection [12, 227–230]. In recent studies, Abs from hybridomas with the ability to cross-neutralize SARS-CoV and SARS-CoV-2 in vitro have been identified [12, 227, 229], similar to those previously reported in terms of cross-neutralization amongst different CoVs (SARS-CoV, MERS-CoV, and SARS-CoV-2) [3, 29, 135, 231, 232].

The mAb 47D11 obtained from SARS-S hybridoma supernatants has been humanized [12]. Importantly, mAb 47D11 does not interfere with the recognition and binding of the S protein with hACE2, owing to a mechanism that remains unknown [12, 47]. In addition, it has been demonstrated that its ability to perform a cross-neutralization is possibly by interactions with the conserved central region of the S protein from the RBD domain [12] (Additional file 1: Table S3). Another Ab obtained by the hybridoma technique is MAB362, whose variable sequences are expressed as IgG or monomeric IgA isotypes. IgA-type Ab presents higher neutralizing activity than its IgG homolog due to its “longer arms” and “greater flexibility” in the hinge domain, which allows the neutralization of S proteins from other CoVs. Additionally, IgA is proposed to have a greater persistence in mucosal secretions compared with the other isotypes [227]. Another mAb obtained from hybridoma cells is mAb#11/9, which binds the S protein irrespective of its glycosylation pattern [228], being also recognized by Abs SiD7h and S3D8h (IC_50_ of 113.3 ng/mL and 137.2 ng/mL, respectively) [229]. Furthermore, six groups of mice hybridoma Abs with neutralizing capacity that recognized different epitopes on RBD has been described and can be employed as diagnostic tools in SARS-CoV-2 infection [230]. Using the same technique, the Abs 2H2 and 3C1 were developed, targeting different regions of S. Both have the ability to neutralize infection by SARS-CoV-2 virus. In particular 2H2, potently neutralize SARS-CoV-2 pseudovirus (IC_50_: 0.025 μg/mL) and authentic SARS-CoV-2 (IC_50_: 0.007 μg/mL). Interestingly, the human–mouse chimeric Abs c2H2 and the c2H2/c3C1 cocktail (with the same IC_50_ of 0.054 μg/mL) could significantly reduce viral loads in Balb/c mice, showing therapeutic efficacy [210].

### Neutralization of SARS-CoV-2 by nanobodies

In mammals, Abs present two chains (heavy and light), whereas in camelids, Abs containing homodimeric heavy chain with no C_H_1 but a conserved Ag-binding domain (V_H_H) can be found (Fig. 2e). The V_H_H is also known as a single-domain Ab (sdAb) or nanobody (Nb) (Fig. 2d, f), which can be selected from synthetic, naive, or immunized cDNA libraries using phage, bacterial, yeast, or ribosomal display technologies [233–235]. Nbs have the smallest structures (~ 13 kDa) compared with other Abs, present with antigenic recognition, can act in a monomeric form or fusion protein, and show high specificity, stability, and solubility [236]. Therefore, Nbs are valuable in biomedical research. The first therapeutically active Nb caplacizumab-yhdp designed for treating thrombotic thrombocytopenic purpura and thrombosis (Ablynx, a Sanofi Company, Ghent, Belgium) was approved by the European Medicines Agency (EMA) in 2018 and the U.S. Food and Drug Administration (FDA) in 2019 [237]. Accordingly, Nbs with high affinity against SARS-CoV-2 S proteins, and the RBD could emerge as potential therapeutics in the fight against COVID-19, in line with the repertoire of potent neutralizing Nbs previously reported (Additional file 1: Table S5).

#### Camelid immune libraries

Distinct Nbs have been developed against SARS-CoV and MERS-CoV, such as V_H_H-55 and V_H_H-72 [238]. V_H_H-72 when converted into a bivalent Fc (human IgG1) fusion form neutralizes the S protein of SARS-CoV-2 pseudovirus (IC_50_ of ~ 0.2 µg/mL). Pretreatment with V_H_H-72-Fc has been observed to reduce the viral load in the lungs of Syrian hamsters by ~ 10^5^-fold compared with that in the untreated control animals [239] (Additional file 1: Table S5). According to the differing neutralization effects noted for V_H_H-72 and V_H_H-72-Fc, there exists different epitopes between them, and the crystal structures indicate that V_H_H-72-Fc interacts with the RBD as a Class 4 Ab [240]. ExeVir Company (Ghent, Belgium) has advanced with the development of V_H_H-72-Fc through preclinical and clinical trials. The NIH-CoVnb-112 Nb has been obtained from a llama immunized with the SARS-CoV-2 S protein, and it blocks the SARS-CoV-2 RBD and hACE2 engagement [240]. W25UACh obtained from a V_H_H library using *E. coli* display, is able to recognize beads coated with the S protein [241]. Ty1 binds the RBD with a high affinity and present neutralization activity against SARS-CoV-2 pseudovirus (IC_50_: 77 ng/mL), avoiding hACE2 interaction; cryo-EM reconstruction revealed this complex with the RBD in both the “up” and “down” conformations as belonging to Class 2 having Abs with a quaternary epitope [242] (Additional file 1: Table S5). Ty1 multimeric constructs as the tetramer 4-arm PEG Ty1 increased its neutralizing capacity dramatically (IC_50_: 13 pM) [243].

In contrast, Nbs such as Nb-Set1, NM1226, NM1228, NM1230, and NM1224, also derived from an immunized camelid, present high neutralization potencies against authentic SARS-CoV-2 (IC_50_: ~ 15 nM, ~ 7 nM, ~ 37 nM, and ~ 256 nM, respectively). Furthermore, these Nbs block the RBD-hACE2 interaction and target different epitopes within the RBD [244]. Moreover, the Nbs 89, 20, and 21 obtained from the RBD-immunized camelid serum present high neutralization activities against authentic SARS-CoV-2 (IC_50_: 20.154 nM, 0.048 nM, and 0.22, respectively) [237] (Additional file 1: Table S5). By modeling the Nbs 20 and 21, it has been revealed that they probably interact with the RBD in the “down” conformation [245]. On the other hand, Nb11-59, was obtained from camels immunized with the recombinant RBD of SARS-CoV-2, and present neutralizing activity against the authentic SARS-CoV-2 with neutralizing dose 50 (ND_50_) of 0.55 μg/mL, and inhibit the replication of eight RBD SARS-CoV-2 mutants (Q321L, V341I, N354D, V367F, K378R, V483A, Y508H, H519P) [246].

#### Camelid naïve libraries

H11 has been identified in a naïve llama V_H_H library using phage display and found to target the RBD. Later, using random mutagenesis, Nbs H11-D4 and H11-H4 have been generated. Both Nbs are able to block the attachment of the S protein to hACE2 in vitro, recognizing different epitopes compared with CR3022 [30]. H11-D4-Fc and H11-H4-Fc fusions show neutralizing activity against authentic SARS-CoV-2 (ND_50_: 18 nM and 6 nM, respectively) [30]. Cryo-EM reveals that both Nbs bind RBDs in the “up” and “down” conformations in the S protein trimer and hence, these are categorized as Class 2 Abs [30].

Three synthetic V_H_H camelid libraries using ribosome and phage displays have allowed the generation of synthetic Nbs, termed as “sybodies” (Sbs) [247]. Sixty-three anti-RBD Sbs have been obtained from three libraries, screened by one round of ribosome display and two rounds of phage display against the RBD [248]. Other Sbs have been isolated from libraries such as SR4, MR17, MR3, and MR4, presenting high neutralization potencies against SARS-CoV-2 pseudovirus (IC_50_: 5.9, 12.32, 0.40, and 0.74 µg/mL, respectively). Crystal structures of the complexes of Sbs and the RBD reveal a common neutralizing mechanism, suggesting that SR4, MR17, and probably MR3 interfere with the interaction between the RBD and hACE2 [249]. Divalent-engineered Sbs used to synthetize the MR3-MR3-albumin binding domain have been demonstrated to be the best for improving neutralization activities against pseudotyped virus (IC_50_: 0.012 µg/mL). These Sbs have also been evaluated in SARS-CoV-2-infected C57BL/6J female mice, and the lung viral titers were found to be 50-fold lower than that in the control mice [249]. In the Sb-treated group, the alveolar wall structures were normal, although mild bronchopneumonia was observed; whereas, the lung viral load was reduced [249]. Sb23 has been isolated from a synthetic library, and interferes with the RBD and hACE2 interaction showing neutralizing activity against SARS-CoV-2-S pseudotyped virus (IC_50_: 0.6 µg/mL) [250]. The cryo-EM structure suggested Sb23 as a Class 2 indicating that interacts with the S protein, wherein two RBDs are in the “up” conformation [250]. SR31, another Sb isolated from a synthetic library, interacts with the RBD, distorting it, and does not neutralize SARS-CoV-2 pseudovirus [251]. Since SR31 displays high affinity, its fusion with other neutralizing Sbs, such as SR31-MR17 or SR31-MR6, increases the neutralization activity against SARS-CoV-2 pseudovirus (IC_50_: 52.8 µg/mL or 2.7 µg/mL, respectively) [251].

The synthetic Nbs Nb3, Nb6, and Nb11 have been obtained through screening of a yeast surface-displayed library using multiple S protein epitopes [252]. Particularly, Nb6 has a potent neutralization activity against pseudovirus infection (IC_50_: 2.0 µM). Cryo-EM revealed that Nb6 binds to the RBD in the open and closed S conformations, and belongs to Class 2 according to the Barnes classification [194]. Furthermore, the trivalent version of Nb6 (Nb6-tri) has shown an improvement in the neutralization activity against the authentic SARS-CoV-2 (IC_50_: 140 pM) [252]. In addition, multi-specific V_H_H Abs fused to human IgG1 Fc domains are able to activate the Fc-dependent functions, such as tri-specific V_H_H-Fc 3F-1B-2A, which has been designed to neutralize SARS-CoV-2 [253]. The tri-specific V_H_H-Fc 3F-1B-2A Ab, which in a docking model has been observed to interact with the RBD, exhibits higher pseudovirus neutralization activity than that by other V_H_H-Fc combinations (IC_50_: 3.0 nM) [253] (Additional file 1: Table S5). Other Nbs have been obtained from camelid V_H_H naïve and synthetic libraries. Nbs were fused with IgG1 Fc domains to obtain two monoclonal V_H_H-Fc named as 1B and 3F and one bi-specific 1B-3F-Fc, which block the binding of hACE2 with S, being 1B-3F-Fc the best in blocking function [254]. In the same sense, the Nb nanosota-1C was obtained from naïve camelid Nb phage display library, which presents high RBD affinity. The same Nb in Fc format (Nanosota-1C-Fc) increases the RBD affinity and presents strong neutralizing effect against SARS-CoV-2 pseudovirus (ND_50_: 270 ng/mL) and against authentic virus (ND_50_: 160 ng/mL). Interestingly, it protected prophylactically, and therapeutically Syrian hamsters infected with SARS-CoV-2 [255].

#### Human Nbs against SARS-CoV-2

Humanization of camelid Nbs has been aimed to reduce their immunogenicity. Nbs against the SARS-CoV-2 RBD have been detected in a library of phage-displayed sdAbs that uses naïve CDR regions together with human germline frameworks, with varied arrangements [256]. Among them, n3088 and n3130 inhibit SARS-CoV-2 pseudovirus infection (IC_50_: 3.3 and 3.7 µg/mL, respectively), and neutralize the authentic SARS-CoV-2 (IC_50_: 2.6 and 4.0 µg/mL respectively) [256]. Both n3088 and n3130 share some residues with the cryptic epitope recognized by CR3022, as suggested by binding models [28, 256]. Another study identified V_H_ ab8 fused with human IgG1 Fc (V_H_-Fc ab8), and this bivalent form shows a potent neutralization activity against pseudotyped SARS-CoV-2 (IC_50_: 0.03 µg/mL), as well as the authentic SARS-CoV-2 (IC_50_: 0.04 µg/mL). V_H_-Fc ab8 is categorized as Class 2 Ab, and it competitively inhibits the hACE2-RBD interaction by occupying three RBDs (two in the “down” and one in the “up” conformation) [209]. Furthermore, V_H_-Fc ab8 binds several RBD mutants found in patients with COVID-19, and its prophylactic and therapeutic efficacy has been demonstrated against SARS-CoV-2 infection in hamsters [209].

Other synthetic humanized sdAbs (1E2, 2F2, 3F11, 4D8, and 5F8) in a bivalent form fused with human IgG1 Fc have been shown to inhibit the association between the RBD and hACE2, presenting superior neutralization potencies against pseudotyped SARS-CoV-2-S (EC_50_: 0.54, 0.40, 0.01, 0.46, and 0.05 µg/mL, respectively) (Additional file 1: Table S5) [257]. Moreover, from a library of engineered human V_H_s, V_H_ ab6 and V_H_ m397 have been obtained, which compete with the RBD for hACE2 binding. Both V_H_s fused with Fc (VH-Fc ab6 and VH-Fc m397) have been shown to neutralize the authentic SARS-CoV-2 (IC_50_: 0.35 µg/mL and 1.5 µg/mL, respectively) and present differences in competition probably due to different target S protein epitopes [258]. Biparatopic and trivalent Nbs against RBD were obtained from human VH-phage library. VH monomers were used to design bi-paratopic or multivalent VHs, with the aim to recognized different RBD epitopes simultaneously [179]. New formats improved the viral neutralization, being the most potent the trivalent VH3 B01 against over authentic SARS-CoV-2 (IC_50_: 3.98 nM), which seems to block simultaneously the hACE2 and RBD interaction through the attack of three RBDs [179].

The diversity of Nbs found and the variety of neutralization mechanisms indicate their promising application in the therapy or prophylaxis of SARS-CoV-2 infection.

## Remarks on the production of mAbs for COVID-19 treatment

Antibodies obtention from patients with COVID-19 is a fruitful strategy to recover human specialized neutralizing Abs. However, few discussions have been conducted on mAb production technologies in order to obtain quality mAbs that are safe, efficient, and accessible to the population [259]. The tetrameric nature of an IgG molecule and its glycosylation is essential for its functioning, making it a challenging protein for expression [260]. In this sense, mammalian cells, such as CHO cells, have become one of the most widely used cell factories for the industrial production of mAbs [261, 262] and are considered the workhorse of the industry [263–265]. Among the 68 mAbs approved between 2014 and 2018, 84% were produced in CHO cells and 16% in cells derived from myelomas (13% in NS0 and 3% in Sp2/0) [262]. Even during the pandemic in 2020, 10 Ab therapeutics had been approved by EMA or FDA [259]. Furthermore, over 60 previously known Abs are under evaluation for possible COVID-19 treatments [259]. Compared with bacteria and yeasts, the yields and productivities of processes based on mammalian cells are low due to the slow rate of cell growth, their tendency to undergo apoptosis, and a low production capacity per cell [260, 261]. Therefore, developing cells with superior production characteristics has been aimed in the field [264, 266–268]. Nevertheless, owing to cell engineering, the time for the establishment of productive cell lines of fully humanized mAbs has sharply reduced, limiting it to some months, with increased productivities (up to 100 pg/cell day, representing bioreactor titers of nearly 10 g/L), which is presently crucial for the production of anti-SARS-CoV-2 mAbs [264, 269–271]. Elevated productive-mAb titres have also been achieved by extensive improvements in the production schemes [272]. Similarly, improvements in the recovery and purification of mAbs have achieved yields of up to 80% of that produced in bioreactors, and consequently, the manufacturing costs of goods have dropped to 20–100 US$ per gram of the active pharmaceutical ingredient [270, 273].

Evidently, with the search for tools to attend the COVID-19 pandemic, and since the start-up of production of mAbs at industrial scale can take at least 6 months, Nbs production became an alternative. Due to the fact that Nbs are smaller and not glycosylated proteins they can be produced in cell factories such as bacteria or yeasts, at lower costs, with a larger scale of production [274]. Moreover, monomeric or multimeric V_H_Hs can be produced without implying major changes in the bioprocess unit operations. However, as they are new molecules with complex and novel structural characteristics, the quality, safety and efficacy tests must be highly rigorous, and the regulatory approval could be longer and intensive than a complete mAb [274, 275]. The humanized Nb11-59 (Additional file 1: Table S4) was expressed in *Pichia pastoris* in small-scale and in 7 L bioreactor, reaching almost 20 g/L of the Nb. HuNb11-59 was also purified by affinity chromatography and hydrophobic chromatography reaching around 95% purity [246].

### CHO cells as producers of anti-SARS-CoV-2 mAbs

CHO cells were established and considered “immortal” by the end of the 1950s [264]. CHO cells present several advantages over other cell types for the production of mAbs: (i) a capacity to perform complex post-translational modifications (PTMs), such as “human”-like glycosylations, protein processing (e.g., phosphorylation) and folding, (ii) robust cell culture in chemically-defined and serum-free media that facilitates scaling-up, (iii) a safe host with a high rate of regulatory approval, and (iv) optimized transfection/selection systems that enable stable expression of heterologous genes [261, 263–265, 276].

Efforts have been made to optimize recombinant protein production in CHO cells, reduce manufacturing costs, and increase accessibility of life-changing drugs to patients [264, 266]. In this sense, mAb production involves a series of processes dictated by transcription (strength of the promoter, integration site for the gene of interest, and mRNA turnover), but also by the translation rate, protein turnover, and protein folding and processing [266]. Limited studies indicate the expression of recombinant anti-SARS-CoV-2 mAbs in CHO cell lines [145] some using transient expression [17, 178, 184, 277] with low utility at an industrial scale. Moreover, there are some studies on the production of the His-tagged SARS-CoV-2 S protein trimer or the S protein by CHO cells [27, 278]. Furthermore, to date there are at least 21 clinical studies evaluating anti-SARS-CoV-2 mAbs (Additional file 1: Table S4); however, information of large-scale production of mAbs has not been reported [52, 53, 279].

### Challenges in the production of anti-SARS-CoV-2 mAbs

Although there are more than 70 mAbs licensed, only few of them have been approved or are under review in the EU or USA to treat or prevent diseases caused by viruses, such as human immunodeficiency virus infection, lower respiratory tract disease caused by syncytial virus in children, Ebola, and recently, COVID-19 [52, 53, 259]. Moreover, there are just one therapeutically active Nb approved in USA and EU [237]. Some of the mAb products are a combination of two or three mAbs. Accordingly, therapeutic Abs against SARS-CoV-2 should be combined cocktails that they recognize different epitopes in S including varied Abs formats and classes [52, 53, 144, 280]. Hence, the current challenges in large-scale production of Abs necessary to fight the pandemic are related to achieving production of all formats of Abs in cell factories with elevated productivities, as well as the scaling-up bioprocesses to generate enough amounts of the active pharmaceutical ingredient (API) to cover the world population.

Moreover, the location and optimum operation of production plants with stringent quality control practices worldwide, including Africa and Latin America, needs to be determined, along with the recruitment of skilled labor. Furthermore, multiproduct facilities should be considered to produce Ab cocktails. In addition to the quality assurance, it is necessary to demonstrate batch-by-batch reproducibility in bioprocesses. The production of anti-SARS-CoV-2 mAbs in CHO cells can be seen as a feasible strategy for implementation on an industrial scale in conjunction with high-density cultures [272], single-use technologies [267], design/selection techniques for highly productive clones [264, 281], and bioprocess optimization [266]. To achieve this, it is necessary that companies with large biotechnological developments provide insights to the bio-pharmacological industry to combat this global pandemic.

### Upstream and downstream bioprocesses in mAb production

**Fig. 6:**
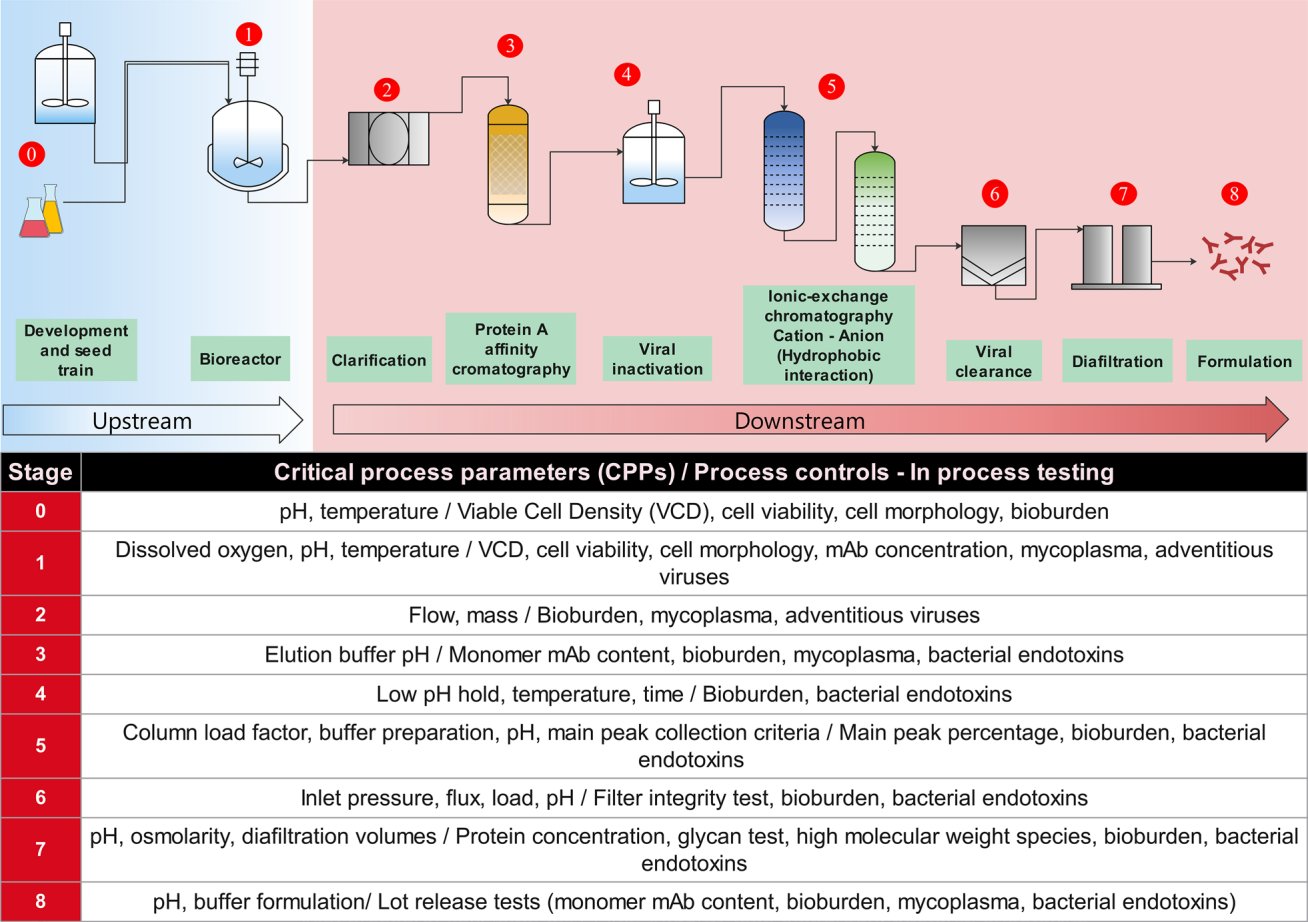
A proposed simplified bioprocess flow diagram for an anti-SARS-CoV-2 monoclonal antibody production: 0. Inoculum train and culture media preparation. 1. Production bioreactor. 2. Cell harvesting (centrifugation or filtration). 3. Affinity (Protein-A) purification. 4. Low pH viral inactivation. 5. Ion exchange chromatography. 6. Virus removal. 7. Ultrafiltration / diafiltration and 8. Active Pharmaceutical Ingredient (API) formulation

The upstream production of mAbs in CHO cells is widely conducted using suspension cultures in stirred tank bioreactors under different modes: batch, fed-batch, and perfusion (continuous culture with retention of cells), although in the process of increasing the cell number (inoculum train) to reach the production volume, other types of bioreactors are also used, such as shake flasks and wave bioreactors, with preference for single-use systems [267, 282]. While, the production Nbs bioprocesses can be carried out in bacterial or yeasts conventional biopharmaceutical plants, where the inoculum train and the production bioreactors are generally made of stainless steel, with economical culture media, implying larger industrial scales than those of animal cells. In general, upstream process development also includes scaling-up the culture process for reproduction on a large scale. An in-depth understanding of the process and its critical process parameters (CPPs) is essential to achieve a successful scale-up. The scale-up strategy involves keeping one or two parameters constant (scale-up criterion) across the different production scales [283]. In the case of mammalian cells, the aim is usually to keep the shear forces or oxygen transfer constant [283]. Moreover, technological, analytical, and regulatory advances promote biopharmacies to implement continuous culture systems to meet the growing demand for mAb production [275, 282, 284]. mAb isolation and purification (downstream) represent a significant portion of the production effort and costs [270, 285]. Hence, improvements in these aspects are relevant [285]. Furthermore, the downstream mAb manufacturing processes do not have a standard framework (Fig. 6); all processes rely on biomass clarification (biomass removal) and protein-A chromatography as the initial capture steps (providing in some cases > 98% purity in a single purification step). Subsequently, low-pH viral particle inactivation, viral particle removal, and polishing chromatographic steps are used to obtain the API [270]. The Nbs downstream can follow the procedures typically used for human therapeutic recombinant proteins (such as hormones, cytokines or stimulatory factors) produced in bacteria and yeast [246, 286].

The production level and the quality of Abs are highly sensitive to the operating conditions of the production process [287]. Ab production implies identification of the critical stages and variables in the process (Fig. 6), as well as determination of the critical quality attributes (CQAs) of the Ab to ensure its quality, safety, and efficacy (Additional file 1: Table S6). Then, CQAs are physical, chemical, biological, or microbiological properties with defined statistical limits, ranges, or distributions [287] (Additional file 1: Table S6). Given the relevance of the CQAs, the concept of quality by design (QbD) emerges as an experimental strategy that requires a deep understanding of the bioprocess to determine the CPPs and the in-process testing parameters of the unit operations (Fig. 6) [287]. The implementation of process analytical technologies (PAT) is the most helpful strategy to ensure the quality, safety, and efficacy of mAbs [282]. CQAs should be carefully controlled and measured during both, upstream and downstream bioprocesses (Additional file 1: Table S6). Finally, the CQAs for an Ab finished pharmaceutical product must be taken into account, such as the pH, protein amount, formulation, freezing/thawing, dose, color, lyophilization, drug delivery, and logistics, all of which, depend on the final product presentation.

## Conclusions and perspectives

mAbs have revolutionized the treatment of different diseases, including viral diseases. Their applications in human therapy have improved with the development of completely human mAbs, thereby ensuring their quality, efficacy, and safety with reduced immunogenicity. However, the cost of Abs has always been a limiting factor in their use in the clinical settings, as well as their large-scale distribution. This limitation has been overcome by improving the production processes, as well as the purification strategies.

Until now, different studies have shown that the immune response in many COVID-19 patients leads to the production of CP, which contains specialized neutralizing Abs that can protect the patient against SARS-CoV-2, in conjunction with a series of other favorable immune responses. The recovery of the genetic and protein information on the Abs produced in COVID-19 patients and other immunized animals has allowed the identification of hundreds of Abs with neutralizing activity. Furthermore, these discoveries have led to the expression and production of Abs in different formats (mAbs, Fabs, Nbs, and sdAbs) to be characterized physically, chemically, and structurally. Some of these Abs are being tested in animal models, undergoing clinical trials, or recently mAbs approved for emergency use in humans. A cascade of information has been generated in this regard, which will surely lead to the generation of therapeutic and prophylactic solutions for the treatment of COVID-19, an unprecedented disease that remains uncontrolled globally. Thus, the search for neutralizing Abs that can serve in the control and therapy of SARS-CoV-2 infection needs to be exhaustive and remains urgent.

In COVID-19 patients, neutralizing Ab titers are correlated with the severity of the infection [12, 128, 178] and low somatic hypermutation [137, 138, 142, 180]. The RBD region is found to be immunodominant and the target of approximately 90% of the neutralizing Abs present in the sera of SARS-CoV-2-infected people. Furthermore, it has been determined that the anti-RBD IgG titers decrease with time post symptom onset, presenting a half-life of approximately 49 days. Importantly, avidity increases over time, probably due to increased maturation. In the serum of hospitalized COVID-19 patients, there is a greater number of IgG against the protein S and the RBD, compared with that in non-serious and asymptomatic patients [178]. Therefore, all patients present anti-RBD IgGs, from patients with severe (most of them older than 50 years), high, medium, low, or atypical symptoms to asymptomatic patients (diverse age group with most of them between 20 and 60 years of age). The pediatric antibody production is different, since they produce anti-S but not anti-N Abs and present untrained T-cell responses together with a strong immune response acquired at birth that allows faster virus elimination [192, 288, 289]. In contrast, hospitalized adult patients present a high titer of Abs that block the interaction between the RBD and hACE2 [178]. Therefore, CP therapy using plasma from adult patients could increase the Ab content, and accordingly, it has been used as treatment in different clinical trials [147, 152, 153]. However, many concerns around the safety and efficacy of CP against COVID-19 exist. Consequently, the possibility of identifying neutralizing Abs and their characterization will avoid application of the whole CP.

Hence, in-depth analyses, isolation, characterization, and production of neutralizing Abs found in COVID-19 patients would allow for rational proposals of immunological protection [178]. Moreover, generation of polyvalent antivirals with at least four targets, as described by the four different Ab classes, using mAbs, Fabs, multabodies, Nbs, Sbs, or fusion proteins that interact with the RBD in the “up” and “down” conformations could broaden the spectrum of their therapeutic potential and prevent viral escape through mutations. In addition, combination of Abs, targeting different RBD epitopes or the new variants integrated in cocktails [145, 215, 290], can ensure successful COVID-19 therapy. Of note, although recombinant mAbs have generally been used in human therapies for more than 20 years and are generally well tolerated, adverse effects (skin reactions, pyrexia, anaphylaxis even a systemic inflammatory response and ADE reaction, among others) must be well studied and characterized [48, 50, 101, 102, 291]. Thus, passive immunization treatment could be one strategy to treat severe cases, people who do not respond to vaccination or cannot be vaccinated. Therefore, the vast amount of information generated will allow for the development of safe and effective treatments and vaccines for COVID-19, providing the molecular basis for the neutralization of pathogenic CoVs by Abs.

## Supplementary Information

**Additional file 1: Table S1.** Participation of the immune system in the infection by SARS-CoV-2. **Table S2.** Summary of outcomes regarding the use of convalescent plasma from COVID-19 patients. **Table S3.** Binding affinity of monoclonal antibodies that block or neutralize interaction between SARS-CoV-2 and hACE2. **Table S4.** Clinical evaluation of mAbs against SARS-CoV-2. **Table S5.** Binding affinity of nanobodies that block or neutralize interaction between SARS-CoV-2 and hACE2. **Table S6.** Process and product related potential critical quality attributes (pCQAs) to be taken into account for the production of anti-SARS-CoV-2 mAbs to obtain a pure active pharmaceutical ingredient.

## Data Availability

Not applicable.

## Abbreviations

Ab: Antibody
ADCC: Antibody-dependent cell cytotoxicity
ADCP: Antibody-dependent cellular phagocytosis
ADE: Antibody-dependent enhancement
Ag: Antigen
ARDS: Acute respiratory distress syndrome
CDC: Complement-dependent cytotoxicity
CDR: Complementarity-determining region
CHO: Chinese Hamster Ovary cells
CP: Convalescent plasma
cryo-EM: Cryogenic electron microscopy
EC_50_: Half-maximal effective concentration
ND_50_: Neutralizing dose 50
Fab: Fragment antigen-binding
GM-CSF: Granulocyte–macrophage colony-stimulating factor
hACE2: Human angiotensin-converting enzyme
IC_50_: Half maximal inhibitory concentration
IGHV: Immunoglobulin heavy chain variable region
mAb: Monoclonal antibody
MASP-2: Mannan-binding lectin-associated serine protease 2
MERS-CoV: Middle East respiratory syndrome coronavirus
Nbs: Nanobodies
NK: Natural killer
NKG2A: Natural Killer Group Protein 2
NTD: N-terminal domain
PAMPs: Pathogen-associated molecular patterns
PDB: Protein data bank
PMTs: Post-translational modifications
PSO: Days-post symptoms onset
RBD: Receptor binding domain
RBM: Receptor-binding motif
RT-PCR: Reverse transcription polymerase chain reaction
SARS-CoV-2: Severe acute respiratory syndrome coronavirus 2
Sb: Sybody
sdAb: Single domain antibody
TLR: Toll-like receptor
VDJ: Variable (V), diversity (D), and joining (J) genes segments
V-NAR: Variable domain new antigen receptor
V_H_H: Variable domain heavy chain
