# Supplementary material for: Integrative overview of antibodies against SARS-CoV-2 and their possible applications in COVID-19 prophylaxis and treatment
Source: Microb Cell Fact. 2021 Apr 22;20:88. doi: 10.1186/s12934-021-01576-5 (PMC8061467; doi:10.1186/s12934-021-01576-5)
Supplement: Supplementary file 1 — Additional file 1: Table S1. Participation of the immune system in the infection by SARS-CoV-2. Table S2. Summary of outcomes regarding the use of convalescent plasma from COVID-19 patients. Table S3. Binding affinity of monoclonal antibodies that block or neutralize interaction between SARS-CoV-2 and hACE2. Table S4. Clinical evaluation of mAbs against SARS-CoV-2. Table S5. Binding affinity of nanobodies that block or neutralize interaction between SARS-CoV-2 and hACE2. Table S6. Process and product related potential critical quality attributes (pCQAs) to be taken into account for the production of anti-SARS-CoV-2 mAbs to obtain a pure active pharmaceutical ingredient. [file 12934_2021_1576_MOESM1_ESM.pdf]

Additional file 1

Integrative overview of antibodies against SARS-CoV-2 and their possible applications in COVID-19 prophylaxis and treatment

Norma A. Valdez-Cruz [1]*, Enrique García-Hernández [2], Clara Espitia [3], Laura Cobos-Marín [4], Claudia Altamirano [5], Carlos G. Bando-Campos [1], Luis F. Cofas-Vargas [2], Enrique W. Coronado-Aceves [3], Ricardo A. González-Hernández [1], Pablo Hernández-Peralta [4], Daniel Juárez-López [1], Paola A. Ortega-Portilla [3], Sara Restrepo-Pineda [1], Patricio Zelada-Cordero [1], Mauricio A. Trujillo-Roldán [1]*

1. Programa de Investigación de Producción de Biomoléculas, Departamento de Biología Molecular y Biotecnología, Instituto de Investigaciones Biomédicas, Universidad Nacional Autónoma de México, Ciudad Universitaria, Ciudad de México, 04510, México.
2. Instituto de Química, Universidad Nacional Autónoma de México, Ciudad Universitaria, Ciudad de México 04510, México.
3. Departamento de Inmunología, Instituto de Investigaciones Biomédicas, Universidad Nacional Autónoma de México, Ciudad Universitaria, Ciudad de México, 04510, México.
4. Facultad de Medicina Veterinaria y Zootecnia, Universidad Nacional Autónoma de México, Ciudad Universitaria, Ciudad de México, 04510, México.
5. Escuela de Ingeniería Bioquímica, Pontificia Universidad Católica de Valparaíso, Av. Brasil N° 2950, Valparaíso, Chile.

***Corresponding authors**

Programa de Investigación de Producción de Biomoléculas, Departamento de Biología Molecular y Biotecnología, Instituto de Investigaciones Biomédicas, Universidad Nacional Autónoma de México, Ciudad Universitaria, Ciudad de México, 04510, México.

Email: maurotru@biomedicas.unam.mx (MATR); adri@biomedicas.unam.mx (NAVC)

Table S1. Participation of the immune system in the infection by SARS CoV-2.

Response	Mechanism of the immune response	Effect		Activity	References
		Adv	Prot		
Innate	TLRs receptor activation		*	TLR3, TLR7, TLR8, and TLR9 activation, leads to NF-kappa B pathway and of pro-inflammatory cytokines	[61, 62]
	Macrophage activation syndrome	*		Hyperactivation of macrophages resulting in cytokine storm (IL-6, IL-7, TNF, CCL2, CCL3, CXCL10, IL-2 receptor alpha chain)	[6, 63, 64, 66]
	Inflammasome activation	*		SARS-CoV protein ORF8 interacts with NLRP3 inflammasome subunit inducing macrophage activation	[68]
	Excessive macrophage migration	*		Macrophage infiltration in the lung tissue had been observed *postmortem*	[72]
	Decrease of NK cells	*		Decrease of NK cell populations and presence of dysfunctional or exhausted phenotypes had been described in severe cases	[73]
	Complement activation	*		Complement hyperactivation due to MASP-2 activation and neutrophil migration-activation in lung tissue and hypercoagulation.	[78, 79]
	Type I and III Interferon		*	Viral replication inhibited (tested *in vitro*)	[89]
	Type I interferon	*		ORF6, ORF8 and N inhibits type I interferon signaling pathway	[70]
Adaptive	$CD8^+$ T cell	*		Poor response due to the presence of exhausted cells and the decrease of IL-2, IFNγ and granzyme B	[74, 97]
	$CD4^+$ T cell	*		Short term response, due to a decrease in memory cells	[96, 104]
	Antigen-presenting cells	*		Poor induction of immune response by decreased expression of MHCI and MHCII	[74]
	IgG	*		Favoring cellular infection by ADE. Unproven in SARS-CoV-2	[113]
	IgAs	*		Inflammation mediated by the induction of IL-6, IL-8, CCL-2 and GM-CSF synthesis in the lung	[120]
	IgM, IgG, IgA		*	Virus neutralization by recognizing specific viral epitopes	[130, 135]

Adverse: Adv; Protective: Pro; Antibody-dependent enhancement: ADE; Toll-Like receptor: TLR; Tumoral Necrosis Factor: TNF; NLR family pyrin domain containing 3: NLRP3; Mannan-binding lectin serine protease: 2: MASP-2; Interferon gamma IFNγ; Major histocompatibility complex MHC; Granulocyte-macrophage colony-stimulating factor: GM-CSF; Nuclear factor-kappa B: NF-kappa B.

Table S2. Summary of outcomes regarding the use of convalescent plasma from COVID-19 patients.

Country/City	# of patients	Patient's age	Observed parameters after CPt	% of negative seroconversion	# of days from post-CPt to seroconversion	Observations	Reference
China, Shenzhen	5	36-73	**↑:** PaO_2/FiO_2, RBD specific IgG and IgM, NAbs; **↓:** SOFA, T°, CRP, IL6, procalcitonin.	100	1-12	No control group.	[147]
China, Dongguan, Xiangtan, Xiaolan	4	31-73	**↑:** PO_2, anti-SARS-CoV-2 IgG; **↓:** Anti-SARS IgM.	100	3-22	No control group.	[149]
China, Wuhan	10	34-78	**↑:** Lymphocytes, SaO_2, NAbs; **↓:** CRP, ALT, AST.	100	2-6	Control group, not randomized.	[152]
China, Wuhan	6	28-75	**↑:** Anti-SARS-CoV-2 IgG and IgM; **↓:** Ground glass opacity.	100	1-12	No control group.	[150]
China, Zhengzhou	6	61.5 (median)	NP	100	1-3	Control group, not randomized.	[148]
China	52	70 (median)	NP	87.2	1- 3	Control group, randomized.	[153]
China, Wuhan	138	65 (median)	**↑:** Lymphocytes, RBD and S protein specific IgG; **↓:** CRP, neutrophils.	80	1-14	Control group	[160]
USA, Texas	25	19-77	**↑:** White blood cells, ALT, bilirubin, ferritin; **↓:** CRP, LDH, AST.	NP	NP	No control group.	[296*]
USA, Connecticut Massachusetts	38	63 (mean)	NP	NP	NP	No control group.	[156]
USA, various cities	5000	62.3 (median)	NP	NP	NP	No control group. ClinicalTrials.gov NCT04338360.	[161]
USA, Wisconsin	31	NP	NP	NP	7; IQR 14	No control group.	[162]
USA Seattle	20	29-95	**↑:** PaO_2/FiO_2; **↓:** CRP, T°, FiO_2	NP	NP	Control group.	[158]
USA New York City	39	55 (mean)	NP	NP	NP	Control group, not randomized.	[159]
Italy, Pavia	46	62 (mean)	**↑:** PaO_2/FiO_2; **↓:** Ferritin, LDH, CRP.	93.5	7	No control group.	[155]
Korea, Seoul	2	67-71	**↑:** PaO_2/FiO_2; **↓:** CRP, IL6, leukocytosis, lymphopenia	100	20-26	No control group.	[151]
Iran, Tehran, Qom, Isfahan Yazd	115	23-93	NP	NP	NP	Control group, not randomized.	[157]

↑: increase; ↓: decrease; Convalescent plasma: CP; CP transfusion: CPt; lactate dehydrogenase: LDH; Aspartate aminotransferase: AST; Alanine aminotransferase: ALT; Sequential Organ Failure Assessment score: SOFA; Partial pressure of oxygen: PaO_2; Fraction of inspired oxygen: FiO_2; Neutralizing antibodies: NAbs; C-reactive protein: CRP; Immunoglobulin G: IgG; Immunoglobulin M: IgM; Interleukin 6: IL6; Temperature: Tº; Not presented: NP.

* Additional references at the end of this supplementary material.

Table S3. Binding affinity of monoclonal antibodies that block or neutralize interaction between SARS-CoV-2 and hACE2.

Number	Source	K_D (nM)	IC_{50} μg/ml	Target	Observations	Reference
CR3022 mAb	Convalescent SARS-CoV	6.3	ND_{50} 93 nM (AV-CoV-2)	RBD up	CR3022 binds to SARS-CoV RBD and presents cross-reactivity with SARS CoV-2 RBD	[28, 29, 30]
CR3022 Fab	Convalescent SARS-CoV	115	ND	RBD up	Cross-reactivity with SARS CoV–2 RBD	[28]
4A8 mAb	PMBC´s from patients SARS-CoV-2	0.99	0.39 (AV-CoV-2)<br>EC_{50} 49.0 (PSV-CoV-2)<br>EC_{50} 0.61 (AV-CoV-2	NTD	Did not block the binding of spike proteins to hACE2 receptor and compete with 1M-1D2.	[141]
2M-10B11 mAb	PMBC´s from patients SARS-CoV-2	0.34	EC_{50} 170.0 (AV-CoV-2)	RBD	Compete with CR3022. Did not neutralize authentic SARS-CoV-2.	[141]
0304-3H3 mAb	PMBC´s from patients SARS-CoV-2	2.14	0.11 (AV-CoV-2)<br>EC_{50} 0.04 (AV-CoV-2)	S2	Neutralize AV-CoV-2.	[141]
1M-1D2 mAb	PMBC´s from C-CoV-2	2.04	25.0 (AV-CoV-2)<br>EC_{50} 28.0 (AV-CoV-2)	S1	Low inhibitory capacities	[141]
47D11 mAb	Collection of mAbs anti-SARS-S hybridoma's	10.8 SARS2-S_{ecto}<br>9.6 SARS2-$S1_B$	0.061 (PSV-CoV)<br>0.061 (PSV-CoV-2)<br>0.19 AV-CoV<br>0.57 AV-CoV-2	RBD	Hybridoma supernatants from immunized transgenic H2L2 mice that encode chimeric immunoglobulins. Presents affinity to SARS and SARS-CoV-2.	[12]
311mab–31B5,	PMBC´s from C-Cov-2	ND	0.0338 (PSV-CoV-2)	RBD	Block SARS-CoV-2 RBD-hACE2 interaction and neutralize PSV entry to host cells expressing hACE2.	[18]
311mab–32D4	PMBC´s from C-CoV-2	ND	0.0698 (PSV-CoV-2)	RBD	Block SARS-CoV-2 RBD-hACE2 interaction and neutralize PSV entry to host cells expressing hACE2.	[18]
B38 mAb	Blood from C-CoV-2	70.1	0.177 (AV-CoV-2)	RBD	B38 avoids bronchopneumonia and interstitial pneumonia in COVID-19 virus–infected hACE2 mice.	[146]
H4 mAb	Blood from C-CoV-2	4.48	0.896 (AV-CoV-2)	RBD	Protected partially against SARS-CoV-2 in hACE2 mice model SARS-CoV-2 infected, mild bronchopneumonia was observed.	[146]
B5 mAb	Blood from C-CoV-2	305.0	1.375 (AV-CoV-2)	RBD partial	Neutralizing activity against COVID-19 virus.	[146]
H2 mAb	Blood from C-CoV-2	14.3	1.0 (AV-CoV-2)	RBD partial	Neutralizing activities against COVID-19 virus.	[146]
H4+B38 mAbs	Blood from C-CoV-2	ND	0.3 (AV-CoV-2)	RBD	B38 and H4 recognize different epitopes on RBD although present partial overlap	[146]
P2B-2F6 mAb	B cells from C-CoV-2	5.14	0.05 (PSV-CoV-2)<br>0.41 AV-CoV-2	RBD	Interfere with the hACE2 receptor.	[135]
P2C-1F11 mAb	B cells from C-CoV-2	2.12	0.03 (PSV-CoV-2)<br>0.03 (AV-CoV-2)	RBD	Neutralizing mAb competitive with hACE2, blocking the interaction between RBD and hACE2.	[135]
P2C-1A3 mAb	B cells from C-CoV-2	2.47	0.62 (PSV-CoV-2)<br>0.28 (AV-CoV-2)	RBD	Neutralizing mAb competitive with hACE2, blocking the interaction between RBD and hACE2.	[135]
P2C-1C10 mAb	B cells from C-CoV-2	15.23	2.62 (PSV-CoV-2)<br>11.12 (AV-CoV-2)	RBD	mAb presents moderate competitive activity with hACE2	[135]
BD-368-2 mAb	B cells from C-CoV-2	0.82	0.0012 (PSV-CoV-2)<br>0.015 (AV-CoV-2)	RBD "up/down"	Blocks the engagement of hACE2, changes the S trimer contributing to its neutralizing activity. Prophylactic efficacy: IP 20 mg/kg mAb 24 h before infection. Therapeutic efficacy: IP 20 mg/kg of mAb injected 2 h after infection into hACE2 transgenic mice.	[32]
BD-218 mAb	B cells from C-CoV-2	0.039	1.1 (PSV-CoV-2<br>0.29 (AV-CoV-2)	RBD	Showed complete viral inhibition.	[32]
BD-395 mAb	B cells from C-CoV-22	0.36	0.020 (PSV-CoV-2)<br>0.27 (AV-CoV-2)	RBD	High potency against both PSV and AV-CoV-2.	[32]
BD-503 mAb	B cells from C-CoV-2	0.24	0.24 nM<br>0.016 (PSV-CoV-2)	RBD	RBD-binding affinity and a neutralizing ability against PSV-CoV-2.	[32]
BD-508 mAb	B cells from C-CoV-2	1.9	1.9 nM<br>0.015 (PSV-CoV-2)	RBD	RBD-binding affinity and a neutralizing ability against PSV SARS-CoV-2.	[32]
BD-515 mAb	B cells from C-CoV-2	0.041	0.022 (PSV-CoV-2)	RBD	RBD-binding affinity and a neutralizing ability against PSV SARS-CoV-2.	[32]
EY6A Fab	PBMC´s from C-CoV-2	2.0	0.39 (AV-CoV-2)	RBD	RBD-binding affinity and a neutralizing ability against AV-CoV-2	[13]

CV07-209 mAb	B cells from C-CoV-2	0.006	0.003 (AV-CoV-2)	RBD	Prophylactic and therapeutic efficacy in golden Syrian hamsters. Therapeutic mAb reduced signs of COVID-19, 1/3 animals presented mild bronchopulmonary, pneumonia and endothelialitis.	[17]
CV07-250 mAb	B cells from C-CoV-2	0.056	0.0035 (AV-CoV-2)	RBD	Reduced hACE2 binding and showed no binding to murine tissue.	[17]
CV07-270 mAb	B cells from C-CoV-2	ND	0.0823 (AV-CoV-2)	RBD	Did not reduce hACE2 binding, and showed binding to smooth muscle tissue	[17]
2-15 mAb	B cells from C-CoV-2	ND	0.005 (PSV-CoV-2)<br>0.0007 (AV-Cov-2)	RBD	Neutralizing antibody with high potency against AV-CoV-2	[205]
1-57 mAb	B cells from C-CoV-2	ND	0.009 (PSV-CoV-2)<br>0.008 (AV-CoV-2)	RBD	Neutralizing antibody with high potency against AV-CoV-2	[205]
2-7 mAb	B cells from C-CoV-2	ND	0.010 (PSV-CoV-2)<br>0.003 (AV-CoV-2)	RBD	Neutralizing antibody with high potency against AV-CoV-2	[205]
5-24 mAb	B cells from C-CoV-2	ND	0.013 (PSV-CoV-2)<br>0.008 (AV-CoV-2)	NTD	Neutralizing antibody with high potency against AV-CoV-2	[205]
HbnC3t1p1 _C6 mAb	B cells from C-CoV-2	0.19	EC_{50} 0.06 (AV-CoV-2)	RBD	Neutralizing antibody that blocks authentic viral infection. Option for prevention and treatment of SARS-CoV-2 infection.	[137]
HbnC3t1p1 _F4 mAb	B cells from C-CoV-2	0.26	EC_{50} 0.04 (AV-CoV-2)	RBD	Neutralizing antibody that blocks authentic viral infection. Option for prevention and treatment of SARS-CoV-2 infection.	[137]
MnC2t1p1_ A3 mAb	B cells from C-CoV-2	0.7	EC_{50} 0.05 (AV-CoV-2)	RBD	Neutralizing antibody that blocks authentic viral infection. Option for prevention and treatment of SARS-CoV-2 infection.	[137]
MnC2t2p1_ C11 mAb	B cells from C-CoV-2	0.02	EC_{50} 0.02 (AV-CoV-2)	RBD	Neutralizing antibody that blocks authentic viral infection. Option for prevention and treatment of SARS-CoV-2 infection.	[137]
CC6.29 mAb	B cells from C-CoV-2	1.2	0.002 (PSV-CoV-2)<br>0.0071 (AV-CoV-2)	RBD-A	mAb exhibited a potent neutralization against SARS-CoV-2.	[16]
CC6.30 mAb	B cells from C-CoV-2	1.71	0.013 (PSV-CoV-2)	RBD-A	mAb exhibited high neutralization against SARS-CoV-2.	[16]
CC6.33 mAb	B cells from C-CoV-2	257	0.039 (PSV-CoV-2))	RBD-B	mAb exhibited a high neutralization, neutralize SARS-CoV-1 (IC_{50} of 162 ng/ml).	[16]
CC12.1 mAb	B cells from C-CoV-2	5.92	0.019 (PSV-CoV-2)<br>0.022 (AV-CoV-2)	RBD-A	Neutralizing antibody. 16.5 mg/kg or 4.2 mg/kg, tested in Syrian hamsters with SARS-CoV-1 infection; no weight loss was observed *vs* controls.	[16]
CC12.3 mAb	B cells from C-CoV-2	5.92	0.018 (PSV-CoV-2)<br>0.026 (AV-CoV-2)	RBD-A	mAb exhibited high neutralization against SARS-CoV-2.	[16]
COV2-2196 mAb	B cells from C-CoV-2	ND	0.0007 (PSV-CoV-2)<br>0.015 (AV-CoV-2)	$S2P_{ecto}$ open	Blocks the engagement of hACE2. Prophylactic efficacy in rhesus macaques (50 mg/Kg) and mice (200 μg per mouse) which developed less lung disease. Therapeutic (20 mg kg^{-1}) efficacy in mice.	[143, 208]
COV2-2130 mAb	B cells from C-CoV-2	ND	0.0016 (PSV-CoV-2)<br>0.107 (AV-CoV-2)	$S2P_{ecto}$ closed	Blocks the engagement of hACE2. Prophylactic efficacy in rhesus macaques (50 mg/Kg) and mice (200 μg per mouse) developing less lung disease. Therapeutic (20 mg kg^{-1}) efficacy in mice.	[143, 208]
COV2-2196/ COV2-2130 mAbs	B cells from C-CoV-2	ND	ND	$S2P_{ecto}$ open and closed	Mice were treated with COV2-2196 and COV2-2130 (developed less lung disease, avoiding weight loss). Therapeutic efficacy of 400 μg per mouse of the cocktail. Infection was neutralized in mice, 12 h after challenge.	[143, 208]
H014 scFv, mAb humanized	Phage display antibody library	ND	3 nM (PSV-CoV-2)<br>38 nM (AV-CoV-2)	RBD	hACE2-humanized mice injected IP 50 mg per kilogram either 4 h after (one dose, therapeutic) or 12 h before and 4 h after (two doses, prophylactic plus therapeutic) with SARS-CoV-2 infection. No lesions of alveolar epithelial cells were observed, indicating potential therapeutic role.	[4, 213]
BD-236 mAb	B cells from C-CoV-2	2.8	0.037 (PSV-CoV-2)	RBD up	Blocks the engagement of hACE2. BD-236 and BD-604 are very similar, only have 4 or 2 amino acid changes in CDRHs and CDRLs, respectively. High-throughput single-cell sequencing	[140]
BD-604 mAb	B cells from C-CoV-2	0.15	0.005 (PSV-CoV-2)	RBD up	BD-604 binds to RBD ~19 fold higher than BD-236 and is more potent against the SARS-CoV-2 pseudovirus, compared to BD-236.	[140]
BD-629 mAb	B cells from C-CoV-2	0.14	0.004 (PSV-CoV-2)	RBD up	BD-629 are different compared to BD-604. However, its neutralization against the SARS-CoV-2 pseudovirus are similar.	[140]

C102 mAb	PMBC´s from C-CoV-2	27.0 (RBD)	0.034 (PSV-CoV-2)	RBD up	Overlaps with the hACE2 binding site. Presents shorth CDRH3s; and could interact with adjacent RBDs, suggesting higher avidity effects.	[142, 194]
C105 mAb	PMBC´s from C-CoV-2	14.0 (RBD)	0.0261 (PSV-CoV-2)	RBD up	Only bind “up” RBD conformation. Shorth CDRH3s. In this class the link of NAbs in adjacent RBDs could achieve increasing the avidity.	[142, 194]
C104 mAb	PMBC´s from C-CoV-2	19.0 (RBD)*	0.0233 (PSV-CoV-2)	RBD “up”/“down”	Quaternary binding different from C144. Was proposed that could interacts between two adjacent down RBD domains or to an “up” RBD.	[142, 194]
C119 mAb	PMBC´s from C-CoV-2	10.0 (RBD)	0.009 (PSV-CoV-2)	RBD “up”/”down”	Quaternary binding with RBD in down conformation adjacent to an “up” RBD or two adjacent down RBD. Binding similar to REGN10987’s.	[142, 194]
C121 mAb	PMBC´s from C-CoV-2	0.5 (RBD)	0.0067 (PSV-CoV-2)<br>0.00164 (AV-CoV-2)	RBD “up”/”down”	Quaternary binding with RBD in down conformation adjacent to an “up” RBD or two adjacent down RBD. Binding similar to REGN10987’s.	[142, 194]
C135 mAb	PMBC´s from C-CoV-2	6.0 (RBD)	0.016 (PSV-CoV-2)<br>0.0029 (AV-CoV-2)	RBD “up”/”down”	C135 Fabs bound with 2 “down” and 1 “up” RBDs (resolved weakly), recognizing the glycosylated epitope N343RBD, without blocking hACE2 engagement.	[142, 194]
C144 mAb	PMBC´s from C-CoV-2	18.0 (RBD)	0.0069 (PSV-CoV-2)<br>0.0025 (AV-CoV-2)	RBD “up”/”down”	Quaternary binding, in the “down” RBD conformation. different from C002, C121, C119, C104.	[142, 194]
414-1 mAb	B cells from C-CoV-2	0.31	3.09 nM (PSV-CoV-2)<br>1.75 nM (AV-CoV-2)	RBD up	Robust viral neutralizing activity.	[214]
553-15 mAb	B cells from C-CoV-2	0.089	1.84 nM (PSV-CoV-2)<br>30 nM (AV-CoV-2)	RBD Epitope B	This antibody could potentiate other antibodies for their neutralizing abilities.	[214]
CA1 mAb	B cells from C-CoV-2	4.68±1.64	ND_{50} 4.65 (PSV-CoV-2)<br>ND_{50} 0.38±0.007 (AV-CoV-2)	RBD	Compete with hACE2; and competes with CB6	[204]
CB6 mAb	B cells from C-CoV-2	2.49±1.65	ND_{50} 0.041 (PSV-CoV-2)<br>ND_{50} 0.036±0.007 (AV-CoV-2)	RBD	Prophylactic and therapeutic protection against SARS-CoV-2 was observed with CB6(LALA) *in vivo* in a rhesus macaque. Reduced the pathological lung damage caused by the infection.	[204]
CV30 mAb	B cells from C-Cov-2	3.63	0.03 (AV-CoV-2)	RBD up	Potentially utility as therapeutic and prophylactic agents to combat the SARS-CoV-2 infection.	[294]
REGN1093 3 mAb	B cells from C-CoV-2, VelocImmune mice	0.041	0.042 nM (S) (PSV-CoV-2)<br>0.037 nM (S) (AV-CoV-2)	RBD up	REGN10933 binds at the top of the RBD, overlapping the hACE2 binding site. ADCC and ADCP activity in primary human cell bioassays utilizing natural killer (NK), mediate ADCC.	[145, 201]
S2E12 Fab	B cells from C-CoV-2	1.6 (RBD)<br>2.5 (S)	5.29 nM (AV-CoV-2)	RBM “up”	The S2E12 bound to three open RBDs on the prefusion SARS- CoV-2 S recognizing the convex RBM tip.	[223]
S2H13 mAb	PBMC from C-CoV-2	149.0 (RBD down)<br>119.0 (S)	0.5 (PSV-CoV-2)	RBD “down”	S2H13 and hACE2 share partially overlapping binding sites to RBD and recognize an epitope that remains accessible in open and closed S states.	[178]
S2H14 mAb	PBMC from C-CoV-2	75.0 (RBD)<br>90.1 (S)	0.9 (PSV-CoV-2)	RBD “up”	S2H14 binding to open RBDs, similar to S230 (Walls et al., 2019) and of the C105 (Barnes et al., 2020).	[178]
C110 mAb	PBMC from C-CoV-2	1.3 (RBD)	0.018 (PSV-CoV-2)	RBD “up”/”down” Class 3	The C110 epitope binding distal to the hACE2 binding motif similar to class 3 and class 2 mAbs. Like REGN10987, could interfere with hACE2.	[194]
COVA1-18 mAb	B cells from C-CoV-2	0.03 (S)<br>0.9 (RBD)	0.008 (PSV-CoV-2)<br>0.007 (AV-CoV-2)	RBD	A strong competition with hACE2 was observed, suggesting blocking ACE2 is it mechanism of neutralization.	[136]
COVA2-04 mAb	B cells from C-CoV-2	2.3 (S)<br>11.2 (RBD)	0.220 (PSV-CoV-2)<br>0.002 (AV-CoV-2)	RBD “up”/”down”	Potent neutralizing mAb, which could block the engagement of hACE2 as a main mechanism of neutralization.	[136]
COVA2-15 mAb	B cells from C-CoV-2	0.6 (S)<br>3.1 (RBD)	0.008 (PSV-CoV-2)<br>0.009 (AV-CoV-2)	RBD “up”/”down”	Potent neutralizing mAb which could block the engagement of hACE2, binding the RBD in up and down conformations.	[136]
COVA2-39 mAb	B cells from C-Cov-2	0.1 (S)<br>1.1 (RBD)	0.036 (PSV-CoV-2)<br>0.054 (AV-CoV-2)	RBD “up”/”down”	Potent neutralizing mAb, showed strong competition with hACE2, binding the RBD in up and down conformations	[136]
REGN1098 7 mAb	B cells from C-CoV-2, VelocImmune mice	0.042 (S)	0.04 nM (S) (PSV-CoV-2)<br>0.042 nM (S) (AV-CoV-2)	RBD “up”/”down” Class 2	REGN10987 bind an epitope located on the side of the RBD, away from the REGN10933 epitope, and has little overlap with the hACE2 binding site. REGN10987 displayed superior ability to mediate ADCC.	[145, 201]
BD23 mAb	B cells from C-CoV-2	ND	4.8	RBD “up”/”down” Class 2	Blocks the binding RBD-hACE2. BD23-Fab is observed per S trimer and it binds the ‘‘down’’ RBD in protomer B. The heavy chain variable domain of is involved in binding to the RBD.	[32]
C002 mAb	PBMC from C-CoV-2	11 (RBD)	0.009 (PSV-CoV-2)	RBD “up”/”down” Class 2	Quaternary binding to “up/down” RBDs like C121, but different to C144. Interaction with RBD in down conformation adjacent to an “up” RBD, probably interacts between two adjacent down RBD domains.	[142, 194]

S309 (Fab)	B cells from C-CoV	0.3 (RBD)<br>~0.2 (S)	0.079 (AV-CoV-2)	RBD down	Block the binding RBD-hACE2, interact and locks the spike in a closed conformation. Bind to adjacent receptor domains as quaternary epitope.	[4, 27, 178]
S304 (Fab)	B cells from C-CoV-2	4.58 (RBD)	>5.0	RBD “down”	The S304 promotes S opening through binding to a Cryptic Epitope in the closed S conformation. Although S304 binds away from the RBM, partial competition between S304 and hACE2 was observed	[27, 184]
S2A4 (Fab)	B cells from C-CoV-2	7.5 (RBD<br>10.0 (S)	3.5 (PSV-CoV-2)	RBD	S2A4 binds to a cryptic epitope requiring opening of two adjacent RBDs, but not overlaps with the hACE2 binding site recognizing an epitope distinct from the RBM. S2A4 could clash with hACE2 within an S trimer.	[178]
LY-CoV555 mAb	PBMCs from C-CoV-2	3.5 (RBD)<br>24 pM (S)	0.012 (PSV-CoV-2)<br>0.020 (AV-CoV-2)	RBD “up”/ “down”	Passive immunization protected from SARS-CoV-2 infection in a rhesus macaque model.	[200]
C1A-B3 Fab	B cells from C-CoV-2	76.3	0.053 (PSV-CoV-2)<br>0.441 (AV-CoV-2)	RBD	A competition with hACE2 binding was observed, blocking the receptor engagement.	[180]
C1A-B12 Fab	B cells from C-CoV-2	4.2	0.081 (PSV-CoV-2)<br>0.062 (AV-CoV-2)	RBD	A competition with hACE2 binding was observed, blocking the receptor engagement.	[180]
C1A-C2 Fab	B cells from C-CoV-2	14.1	0.118 (PSV-CoV-2)<br>0.132 (AV-CoV-2)	RBD	A competition with hACE2 binding was observed, blocking the receptor engagement.	[180]
C1A-F10 Fab	B cells from C-CoV-2	55.7	0.008 (PSV-CoV-2)<br>0.184 (AV-CoV-2)	RBD	A competition with hACE2 binding was observed, blocking the receptor engagement.	[180]
298 (multibody)	VHH-72 fused to light chain of apoferritin	ND	0.00011 (PSV-CoV-2)<br>0.0057 (AV-CoV-2)	RBD	A competition with hACE2 binding was observed, blocking the receptor engagement.	[196]
52 (multibody)	VHH-72 fused to light chain of apoferritin	ND	0.0002 (PSV-CoV-2)<br>0.27 (AV-CoV-2)	NTD	The recognized epitope is partially occluded by NTD, when S is in closed conformation, probably occurring a different mechanism of action.	[196]
P17	phage display antibody library	0.096	165 pM (PSV-CoV-2)<br>0.27 (AV-CoV-2)	RBD “open”	P17 is around 200-fold more potent than H014 against AV-CoV-2. P17 and H014, presents neutralization activity against SARS-CoV, and protective efficacy against SARS-CoV-2 infection in mouse model.	[213]
2G12	Ab that recognizes HIV-1 glycan	317.0	ND	S2 glycan	2G12 binds to the glycan of the S2 protein (observed by cryo-EM), revealing a quaternary epitope.	[199]
STE90-C11	Phage display from C-CoV-2	8.1 (Fab)<br>6.5 (IgG)	0.56 nM (IgG against AV-CoV-2)	RBD	STE90-C11 probably recognize the “open” conformation of the S protein of SARS-CoV-2, specifically, and binds 7 S mutants.	[212]
VIR-7831	B cells from C-CoV	0.021	0.1 (AV-CoV-2)	RBD	Neutralize live wild-type SARS-CoV-2 *in vitro* as well as retain activity against pseudotyped virus variants.	[290]
VIR-7832	B cells from C-CoV	0.021	0.0783 (AV-CoV-2)	RBD	Neutralize live wild-type SARS-CoV-2 in vitro as well as retain activity against pseudotyped virus variants.	[290]
ADI-55689 Fab	Convalescent SARS-CoV	< 10 to SARS-CoV-2 S	EC_{50} 2.03 nM (AV-CoV-2)	S	Cross-reactivity with SARS CoV–2 RBD	[297*]
ADI-56046 Fab	Convalescent SARS-CoV	< 10 to SARS-CoV-2 S	EC_{50} 1.64 nM (AV-CoV-2)	S	Compete with both hACE2 and CR3022, binds slightly farther away from the tip of the RBD.	[297*]

Severe acute respiratory syndrome–coronavirus: SARS-CoV; Respiratory syndrome–coronavirus 2: SARS-CoV-2; Receptor binding domain: RBD; Fresh peripheral blood mononuclear cells: PBMCs; Intraperitoneally: IP; Pseudotyped: PST; Pseudovirus: PSV; Authentic virus SARS-CoV-2: AV-CoV-2; Authentic virus SARS-CoV: AV-CoV; Antibody: Ab; Antigen-binding fragments: Fab; Single-domain antibodies SdAb; Amino-terminus: N-t; Convalescent SARS-CoV-2: C-CoV-2; N-terminal domain: NTD (residues 1-290); Spike ectodomain: S_{ecto}; trimeric S ectodomain: $S2P_{ecto}$; Domain SARS2-S1B: RBD (residues 338–506); Affinity of the indicated Ab and the antigen: K_D; K_D determined from a two-state binding model: * K_D; Apparent affinities: App#1 and #2 (Tortorici et al., 2020); Ab concentration that neutralized 50% of infectivity: IC_{50}; 50% neutralization dose: ND_{50}; Antibody-dependent cellular cytotoxicity: ADCC); Antibody- dependent cellular phagocytosis: ADCP; Neutralizing antibodies: NAbs; Monoclonal antibodies: mAbs; The human immunodeficiency virus type 1: HIV-1.

* Additional references at the end of this supplementary material.

Table S4. Clinical evaluation of mAbs against SARS-CoV-2.

Name	Company	Phase	Clinical Trial ID	Reference
Bamlanivimab (LY-CoV555; LY3819253)	Eli Lilly and Company; Junshi Biosciences	FDA (Emergency Use Authorization), 1, 2, 3, 3, 2/3	NCT04411628; NCT04427501; NCT04497987; NCT04501978; NCT04518410	[53, 203]
Etesevimab (LY3832479, JS016, LY-CoV016)	Junshi Biosciences / Eli Lilly and Company	2	NCT04441918; NCT04441931;	[53, 204]
Bamlanivimab (LY-CoV555)<br>Etesevimab (LY3832479)	AbCellera / Eli Lilly and Company	FDA (Emergency Use Authorization) 3	NCT04427501; NCT04497987; NCT04501978	[53, 204, 298*]
REGN-COV2 (REGN10933/Casirivimab + REGN10987/Imdevimab)	Regeneron	FDA (Emergency Use Authorization) 1/2, 1/2, 3	NCT04425629 NCT04426695 NCT04452318	[145, 201]
Sotrovimab<br>(VIR-7831/GSK4182136) (~S309 antibody).	Vir Biotechnol. / GlaxoSmithKline	2/3	NCT04545060; Activ-3 study	[27, 53]
VIR-7831 + Bamlanivimab (LY-CoV555)	Vir Biotechnol. / Eli Lilly and Company	2	NCT04634409	[290, 299*]
AZD7442 (AZD8895/Tixagevimab + AZD1061/Cilgavimab)	AstraZeneca	3	NCT04507256; NCT04625725; NCT04625972	[53, 143, 208]
Regdanvimab (CT-P59)	Celltrion	1, 2/3	NCT04525079; NCT04593641; NCT04602000	[53]
DXP-593 related to BD-368-2	Beigene	1, 2 pending	NCT04532294; NCT04551898	[53]
BGB-DXP604	Beigene	1	NCT04669262;	[53]
BGB-DXP604 / BGB-DXP593	Beigene	2	NCT04551898; NCT04532294;	[53]
SCTA01	Sinocelltech Ltd.	2/3 pending	NCT04483375; NCT04644185	[4]
TY027	Tychan Pte. Ltd.	3	NCT04429529; NCT04649515	[53]
BRII-196	Brii Biosciences/NIAID	3	NCT04479631; Activ-3 study	[53, 135]
BRII-198	Brii Biosciences/NIAID	3	NCT04479644; Activ-3 study	[53, 135]
BRII-196 + BRII-198	Brii Biosciences/NIAID	2/3	NCT04518410	[53, 135]
ABBV-47D11	AbbVie	1 pending	NCT04644120	[12]
ABBV-47D11 and ABBV-2B04	AbbVie	1	NCT04644120	[12, 27, 53]
COVI-GUARD (STI-1499)	Sorrento Therapeutics, Inc.	1	NCT04454398	[32, 53]
COVI-AMG (STI-2020)	Sorrento Therapeutics, Inc.	2 pending	NCT04734860	[32, 53]
MW33	Mabwell Bioscience Co., Ltd.	1	NCT04533048	[53]
HFB30132A	HiFiBiO Therapeutics	1	NCT04590430	[53]
HLX70	Hengenix Biotech Inc	1 pending	NCT04561076	[53]
ADM03820	Ology Bioservices	1 pending	NCT04592549	[53]
DZIF-10c	U. Cologne / Boehringer Ingelheim	1/2 pending	NCT04631705; NCT04631666	[137]
ADG20	Adagio Therapeutics	1/2/3	NCT04805671	[53]
JMB2002	Jemincare Group	1	NA	[53, 300*]
LY-CovMab	Luye Pharma Group Ltd	1	NA	[53]
C-144-LS and C-135-LS	Bristol-Myers Squibb, Rockefeller University	1	NCT04700163	[53, 142]
COR-101	CORAT Therapeutics	1/2 pending	NCT04674566	[53]
JS016 / LY3832479 / LY3819253	Junshi Biosciences / Eli Lilly & Co.	2	NCT04441918; NCT04441931; NCT04427501	[53, 204]
Anti-SARS-CoV-2 mAb	Stanford University	1		[53]

Monoclonal antibodies: mAbs.

* Additional references at the end of this supplementary material.

Table S5. Binding affinity of nanobodies that block or neutralize interaction between SARS-CoV-2 and hACE2.

Number	Source	K_D	IC_{50}	Target	Observations	Classification[a]	Reference
**Camelid Immune library**							
V_HH-72	Camelid Immune library PhD	38.6 nM (RBD-SD1)	~0.2 µg/mL (PST-CoV-2) (V_HH-72-Fc)	SARS-CoV-1 MERS-CoV SARS-CoV-2 S	V_HH-72 cross reacts with SARS-CoV-1 RBD Bivalent V_HH-72-Fc, recognize a cryptic epitope	Class 4	[238]
V_HH-72-Fc	Camelid Immune library PhD	NA	NA	SARS-CoV-1 MERS-CoV SARS-CoV-2 S	Syrian hamsters inoculated intranasally with original SARS-CoV-2. V_HH-72-Fc was administered IP at 20mg/kg 1 day prior to infection, reducing viral load in lungs ~10^5-fold compared to control animals.	Class 4	[239]
VHH72_S56A-Fc	Camelid Immune library PhD	0.047 nM	0.837 µg/ml (humV_HH_S56A) (PST-CoV-2)	SARS-CoV-2 S	VHH72_S56A-Fc strongly restricted replication of both original and D614G mutant variants in Syrian hamster SARS-CoV-2 challenge model. and minimized the development of lung damage.	NA	[301*]
Ty1	Camelid Immune library PhD	8.0 ± 1.5 nM	0.77 µg/mL (PSV -CoV-2) (Ty1) ~12 ng/mL (PSV -CoV-2) (Ty1-Fc)	RBD	Ty1fusion to Fc domain has extremely potent neutralization activity. 12.8 kDa Nb, recognize a quaternary epitope	Class 2 Quaternary epitope	[242]
tetramer 4-arm PEG Ty1	Camelid Immune library Phage display	NA	013 pM (PSV-CoV-2)	RBD	AV-CoV-2 and PSV-CoV-2 neutralizing assays resulted comparable	Class 2	[243]
NM1226	Camelid Immune library PhD	3.66 nM	0.82 nM (hACE2:RBD inhibition) 1.44 nM (hACE2:S1 inhibition) 0.63 nM (hACE2:Spike inhibition) 15.11 nM (recombinant-CoV-2)	RBD	NM1226, NM1227, NM1228 and NM1229 could not bind simultaneously to RBD, but interfere interaction between RBD-hACE2.	NA	[244]
NM1228	Camelid Immune library PhD	1.37 nM	0.50 nM (hACE2:RBD) 0.85 nM (hACE2:S) 0.32 nM (hACE2:S)	RBD	NM1228 interacted with the RBD at the back/ lower right site. Strong inhibitory effect of hACE2.	NA	[244]
NM1230	Camelid Immune library PhD	8.23 nM	2.12 nM (hACE2:RBD) 10.57 nM (hACE2:S1) 1.96 nM (hACE2:S)	RBD	NM1221, NM1222 and NM1230, probably recognize similar epitope, they cannot bind simultaneously to RBD. Combination like NM1226+NM1230 or NM1228+NM1230, presented virus neutralization.	NA	[244]
NIH-CoVnb-112	Camelid Immune library PhD	4.94 nM	0.323 µg/mL (23.1 nM) (PSV-CoV-2)	RBD	Blocks interaction between hACE2 and RBD.	NA	[240]
W25UACh Monomeric	Camelid Immune library PhD	0.295 ± 0.084 nM	9.28±1.92 nM (AV-CoV-2 D614) 5.09±1.09 nM(AV-CoV-2 G614)	RBD	W25 inhibit the circulating virus containing the S protein D614G mutation	NA	[241]
Nb 89	Camelid Immune library PhD	108 pM	2.1 ng/mL (0.137 nM) (PSV -CoV-2) 0.154 nM (AV-CoV-2)	RBD	Nb 89 Blocks interaction between the hACE2 and RBD, and competes with Nb21, indicating similar epitopes.	NA	[245]
Nb 20	Camelid Immune library PhD	10.4 pM	1.6 ng/mL (0.102 nM) (PSV -CoV-2) 0.048 nM (AV-CoV-2)	RBD	Nb 20 partially overlaps with the hACE2 binding site and can bind the closed S conformation with all RBDs “down”.	NA	[245]
Nb 21	Camelid Immune library PhD	<1 pM	0.7 ng/mL (0.045 nM) (PSV -CoV-2) 0.021 nM (AV-CoV-2)	RBD	Nbs 20 and 21 lock RBDs in their “down” conformation, interfering the hACE2 interaction with RBD in “open” conformation.	NA	[245]
Nb11-59	Camelid Immune library Phage display	21.6 nM	ND_{50}: 550 ng/mL (PSV -CoV-2) 0.021 nM (AV-CoV-2)	RBD	High affinity against RBD SARS-CoV-2 and mutants	NA	[245]
V_HH E	Camelid Immune library Phage display	1.86 nM	60 nM (Vesicular stomatitis virus; PSV, SARS-CoV-2 S Δ18) 48 nM (AV-CoV-2)	RBD	VHH E stabilizes a conformation of the S with all three RBDs in the “up” conformation	NA	[302*]
aRBD-2-5	Camelid Immune library Phage display	59.2 pM	ND_{50}: 1.22 ng/mL (~0.043 nM) (AV-CoV-2)	RBD	Can block RBD-hACE2 interaction	NA	[303*]
aRBD-2-7	Camelid Immune library Phage display	0.25 nM	ND_{50}: 3.18 ng/mL (~0.111 nM) (AV-CoV-2)	RBD	Can block RBD-hACE2 interaction	NA	[303*]
Nb15-NbH-Nb15	Camelid Immune library Phage display	0.54 nM	0.4 ng/ml (AV-CoV-2)	RBD	Exhibited potent inhibitory activity against the wild-type and variants of SARS-CoV-2, including the variant with N501Y mutation. Provided 100% protection against SARS-CoV-2 infection in transgenic hACE2 mice.	NA	[304*]
nAb1 and nAb2	Camelid Immune library Phage display	6 to 15 nM	NA	RBD	Rapid antigen diagnostic kit that detects United Kingdom (UK) and South Africa SARS-CoV-2 variants as well as the RBD of the clinical strain Wuhan-Hu-1.	NA	[305*]

Nb12	Camelid Immune library Phage display	30 nM	11.7 nM (168.5 ng/ml) (PST-CoV-2)	RBD	Llama Nb. Nb12 induce a 2-RBD-up, 1 RBD-down spike conformation, recognizing outside of the hACE2-binding region.	NA	[306*]
Nb19	Camelid Immune library Phage display	4.72 nM	0.335 nM (4.6 ng/ml) (PST-CoV-2)	RBD	Nanomouse and llama Nbs recognize two distinct neutralizing regions on SARS-CoV-2 RBD.	NA	[306*]
**Camelid naïve library**							
Nb H11-D4	Camelid naïve library	39 nM	18 nM (AV-CoV-2; H11-D4)<br>28 nM (hACE2:RBD, H11-D4-Fc)	RBD "up"/ "down"	H11-D4 and H11-H4 recognize the same epitope, overlapping partially with the hACE2.	Class 2 Tertiary epitope	[30]
Nb H11-H4	Camelid naïve library	12 nM	4–6 nM (AV-CoV-2; H11-H4)<br>34 nM (hACE2:RBD; H11-H4-Fc)	RBD "up"/ "down"	H11-D4 and H11-H4 recognize the same epitope, overlapping partially with the hACE2.	Class 2 Tertiary epitope	[30]
Nanosota 1C-Fc	Camelid naïve library	15.7 pM	ND_{50} 0.27 µg/mL (PSV-CoV-2)<br>ND_{50} 0.16 µg/ml (AV-CoV-2)	RBD	Nanosota-1C-Fc was effective preventing and treating hamsters intranasally challenged with SARS-CoV-2	NA	[255]
Nb91-Nb3-hFc	Camelid naïve library	NA	1.54 (PSV-CoV-2)	RBD	Heterodimer Nb presents high RBD affinity than monovalent Nbs, and could be consider as further therapeutic tool.	NA	[307*]
Anti-CS sdAb	Camelid naïve library	26 nM	100ng/mL LV(CoV2-S)	Synthetic peptides of spike (S)	Anti-cleavage site (CS) sdAb blocked the virus infectivity by inhibiting proteolytic processing of SARS-CoV2 S protein	NA	[308*]
**Camelid synthetic library**							
SR4	Camelid synthetic library	14.5 nM	5.90 µg/mL (PSV-CoV-2)	RBD	SR4/MR17 block the interaction between hACE2 with RBD.	Class 2 Tertiary epitope	[249]
MR17	Camelid synthetic library	83.7 nM (MR-17)<br><0.001 nM (Fc-MR17)	12.32 µg/mL (PSV-CoV-2)<br>0.481 µg/mL (PSV-CoV-2) (Fc-MR17)	RBD	MR17-K99Y present a neutralization efficiency comparable to the best Sb MR3.	Class 2 Tertiary epitope	[249]
Sb23	Camelid synthetic library	10.6 nM	0.6 µg/mL (PSV -CoV-2)	RBD	Sb23 binds next to the hACE2 binding site causing steric hindrance for hACE2.	Class 2 Tertiary epitope	[250]
Nb6	Camelid synthetic library	210 nM	2000 nM (PSV-CoV-2 neutralization)<br>3300 nM (AV-CoV-2 neutralization)	Mutant SARS-CoV-2 S*	Binds to the RBD and competes with hACE2. The binding of one Nb6 stabilizes two adjacent RBDs in the "down" conformation.	Class 2 Quaternary epitope	[252]
mNb6	Camelid synthetic library	0.45 nM	6.3 nM (PSV-CoV-2 neutralization)<br>12 nM (AV-CoV-2 neutralization)	Mutant SARS-CoV-2 S*	Mutations I27Y in CDR1 and P105Y in CDR3 increased it affinity by ~500-fold to S* compared to Nb6. mNb6 binds to closed S* ("down" conformation).	Class 2 Quaternary epitope	[252]
mNb6-tri	Camelid synthetic library	<0.001 nM	0.12 nM (PSV-CoV-2)<br>0.054 nM (AV-CoV-2)	Mutant SARS-CoV-2 S*	Binds the RBD and competes with hACE2.	Class 2 Quaternary epitope	[252]
SR31	Camelid synthetic library	5.6 nM	Not neutralizing activity	RBD	SR31 does not inhibit RBD-hACE2 binding and does not neutralize PSV-CoV-2, but recognizes an epitope distant from the RBM.	NA	[251]
MR3	Camelid synthetic library	1.0 nM (MR3)<br>0.22 nM (Fc-MR3)<br>0.22 nM (MR3-MR3 [34 GS])	0.4 µg/mL (PSV-CoV-2) (MR3)<br>0.39 µg/mL (PSV-CoV-2) (Fc-MR3)<br>0.012 (PSV-CoV-2) (MR3-MR3 [34 GS])	RBD	RBD-hACE2 interaction is competitively inhibited by Sbs.	NA	[249]
MR4	Camelid synthetic library	23.3 nM	0.74 µg/mL (PSV-CoV-2)	RBD	Non noticeable neutralization activities.	NA	[249]
MR3-MR3-ADB	Camelid synthetic library	N.A.	4.2 ng/mL (AV-CoV-2) D614<br>5.1 ng/mL (AV-CoV-2) G614	RBD	C57BL/6J mice were injected IP (25 mg MR3-MR3-ADB/kg body weight) after a 12 h AV-SARS-CoV-2 challenge (5x10^{6} $TCID_{50}$). Lung viral titers were 50-fold lower, observing normal alveolar structures.	NA	[249]
Sb#14	Camelid synthetic library	30.75 nM	≥90% reduction at 500 nM (RBD:hACE2 interaction)	RBD-vYFP	Interfered RBD and hACE2 interaction.	NA	[248]
Sb#15	Camelid synthetic library	24.22 nM	≥90% reduction at 500 nM (RBD:hACE2)	RBD-vYFP	Sb#15 and Sb#68 can simultaneously bind to the RBD, are not overlapping binders.	NA	[248]
SR31-MR17	Camelid synthetic library	0.3 nM	52.8 nM (1.65 µg/mL) (PSV-CoV-2)	RBD	SR31-MR17 displayed higher binding affinity compared to SR31 or MR17.	NA	[251]
SR31-MR6	Camelid synthetic library	0.5 nM	2.7 nM (0.08 µg/mL) (PSV-CoV-2)	RBD	SR31-MR6 showed a 27-fold higher neutralization activity compared to MR6.	NA	[251]
V_HH-3F-1B-2A-Fc	Camelid synthetic library	~0.047 nM	0.71 nM (blocking SARS-CoV-2-S)<br>3.00 nM (PSV-CoV-2)	SARS-CoV-2 S1 protein RBD	V_HH-Fcs were able to induce ADCC in Expi293 cells expressing SARS-CoV-2 S.	NA	[253, 254]

V_HH-1B-3F-2A-Fc	Camelid synthetic library	~0.095 nM	0.74 nM (blocking SARS-CoV-2 S)<br>6.44 nM (PSV-CoV-2)	SARS-CoV-2 S1 protein RBD	V_HH-Fcs were able to induce ADCC in Expi293 cells expressing SARS-CoV-2 S.	NA	[253, 254]
n3088	Camelid synthetic library	3.70±0.09 nM (S1)	3.3 µg/ml (PSV-CoV-2)<br>2.6 µg/ml (AV-CoV-2)	SARS-CoV-2 S1 protein RBD	It neutralizes SARS-CoV-2 by targeting a ‘‘cryptic’’ epitope at the S.	NA	[256]
n3130	Camelid synthetic library	55.39±0.98 nM (S1)	3.7 µg/ml (PSV-CoV-2)<br>4.0 µg/ml (AV-CoV-2)	SARS-CoV-2 S1 protein RBD	It neutralizes SARS-CoV-2 by targeting a ‘‘cryptic’’ epitope at the S.	NA	[256]
SR6_c3	Camelid synthetic library	NA	62.7±2.75 nM (PST-CoV-2)	RBD	Six Nbs showed inhibition over PST-CoV-2	NA	[309*]
NbSL18	Synthetic yeast-display Nb library	461±67.5 nM	NA	$PLpro^{CoV-2}$	$PLpro^{CoV-2}$ domain is fundamental for SARS-CoV-2 replication	NA	[310*]
RBD1i13	AHEAD Yeast display	32.2 nM	0.66 nM (0.05 µg/mL) (PST-CoV-2)	RBD	Strong ACE2 competition	NA	[311*]
RBD10i14	AHEAD Yeast display	0.72 nM	5.38 nM (0.42 µg/mL) (PST-CoV-2)	RBD	Reached sub-nanomolar monovalent Kd	NA	[311*]
Sb45	Camelid synthetic library	38 nM	NA	RBD	Sybodies bind RBD with KD values in the nanomolar range.<br>Binds RBD in either up or down position.	Class 2	[312*]
**Human Nb (V_H) library**							
V_H-Fc ab8	Human Nb (V_H) library	0.54 nM	0.03 µg/ml (PSV-CoV-2)	RBD	V_H-Fc ab8 is a potent neutralizer of SARS-CoV-2, that compete with hACE2. (1) BALB/C mice hACE2-adapted SARS-CoV-2 infection, were administered with 36,8 or 2 mg/kg Ab prior to challenge. Neutralized or reduced infection. (2) Hamsters were IP administered 24 h before (prophylaxis) or 6 h after (therapy) virus challenge, decreasing viral RNA and alleviated pneumonia	NA	[209]
V_H-Fc ab6	Human Nb (V_H) library	11 nM	0.35 µg/ml (4.6 nM) (AV-CoV-2)	RBD (residues 330–532)	Compete with hACE2 for binding to the RBD. Neither V_H-Fc ab6 nor V_H-Fc m397 neutralized live AV-CoV.	NA	[258]
V_H3 B01	Human Nb (V_H) library	0.109 nM (Spike-RBD)<br><0.1 nM (Secto)	0.396 ng/mL (PSV-CoV-2)<br>3.98 ng/mL (AV-CoV-2)	RBD	VH domains bounding RBD at the hACE2 binding site	NA	[179]
V_H-Fc m397	Human Nb (V_H) library	9.6 nM	1.5 µg/ml (20 nM) (AV-CoV-2)	RBD (residues 330–532)	Compete with hACE2 for binding to the RBD. V_H-Fc m397 did not neutralized live AV-CoV.	NA	[258]
**Humanized Nb**							
1E2	Humanized Nb	35.52 nM	5.324 nM (PSV-CoV-2)<br>18.47 nM (AV-CoV-2)	RBD	Prevents the binding of SARS-CoV-2 RBD to hACE2	NA	[257]
2F2	Humanized Nb	5.175 nM	0.742 nM (PSV-CoV-2)<br>22.62 nM (AV-CoV-2)	RBD	Partially compete the RBD/hACE2 receptor association	NA	[257]
3F11	Humanized Nb	3.349 nM	0.066 nM (PSV-CoV-2)<br>28.64 nM (AV-CoV-2)	RBD	Partially compete the RBD/hACE2 receptor association	NA	[257]
4D8	Humanized Nb	6.028 nM	0.781 nM (PSV-CoV-2)<br>9.628 nM (AV-CoV-2)	RBD	Completely prevent binding of SARS-CoV-2 RBD to hACE2.	NA	[257]
5F8	Humanized Nb	0.996 nM	0.072 nM (PSV-CoV-2)<br>39.28 nM (AV-CoV-2)	RBD	Partially compete the RBD/hACE2 receptor association.	NA	[257]

Severe acute respiratory syndrome–coronavirus: SARS-CoV; Respiratory syndrome–coronavirus 2: SARS-CoV-2; Receptor binding domain: RBD; Intraperitoneally: IP; Pseudovirus: PSV; authentic virus SARS-CoV-2: AV-CoV-2; authentic virus SARS-CoV: AV-CoV; Convalescent SARS-CoV-2: C-CoV-2; Days post infection: dpi; Recombinant-CoV-2; nanobody: Nb; Sybody: Sb; Spike ectodomain containing S1 and S2: ECD; Not Available: NA; Albumin binding domain: ADB; human ACE2 receptor: hACE2; median tissue culture infectious dose: $TCID_{50}$; Antibody-dependent cellular cytotoxicity: ADCC; Spike protein mutant: S*; Phage display: PhD; 50%Neutralizing Dose: ND_{50}; SARS-CoV-2 papain-like protease: $PLpro^{CoV-2}$.
Classification[a]; according to Barnes et al. [194].

Table S6. Process and product related potential critical quality attributes (pCQA´s) to be taken into account for the production of anti-SARS-CoV-2 mAbs to obtain a pure active pharmaceutical ingredient.

CQA	Risk category	Criticality justification
**Process related CQAs derived from raw material or derived from host cells**		
Residual host cell proteins	Immunogenicity	Host cell derived impurity. Potential immunogenic agent.
Residual host cell DNA	Product safety	Host cell derived impurity. Oncogene transfer potential, safety impact.
Leachables and extractables	Immunogenicity / Product safety	Single use equipment impurity. Safety impact
Leached Protein A	Immunogenicity / Product safety	Impurity derived from the purification process. Immunogenic and mitogenic potential, safety impact
Residual insulin/ antifoams/ antibiotics/methotrexate	Product safety	Impurities derived from the raw material of the bioprocess. Pharmacologically active compounds
Endotoxins / adventitious virus / bioburden / mycoplasm	Product safety	Impurities derived from the raw material and in the bioprocess itself. Safety impact.
**Product related CQA´s**		
Binding to hACE2 receptor	Concentration	HEK / hACE2 - Binding required for mode of action (MoA), required for efficacy, other CQAs could affect.
High molecular weight species (HMW)	Concentration<br>Immunogenicity	Reduced pharmacological activity (binding/potency). Potential immunogenicity risk.
Low molecular weight species (LMW)	Concentration	Reduced pharmacological activity (binding/potency).
Tryptophan oxidation in the complementarity-determining regions (CDR)	Concentration	Oxidation in tryptophans located in the CDRII and CDIII could impact target binding.
Methionine oxidation in the neonatal Fc receptor (FcRn) binding site	Pharmacokinetics (PK)	Methionines located in the CH2-CH3 in the interphase where FcRn binds has a potential impact on pharmacokinetics.
Glycation	Potency	Glycation could reduce binding affinity although Lys residues spread across the molecule. Immunogenicity/safety issues are unlikely due to most human plasma proteins including IgG1 are glycated due to the presence of glucose in serum.
α-Galactosylation	Immunogenicity<br>Safety	Terminal α-(1-3)-galactosylation is reported as immunogenic. Relevant structures like those are not human endogenous structures.
N-Glycolylneuraminic acid (NGNA) sialylated species	Immunogenicity<br>Safety	Glycans with NGNA are reported as immunogenic. Relevant structures like these are not human endogenous structures.

<u>Aditional references (not in reference list on the manuscript):</u>

296. Salazar E, Perez KK, Ashraf M, Chen J, Castillo B, Christensen PA, et al. Treatment of Coronavirus Disease 2019 (COVID-19) Patients with Convalescent Plasma. Am J Pathol. 2020 Aug;190(8):1680-1690. doi: 10.1016/j.ajpath.2020.05.014.
297. Wec AZ, Wrapp D, Herbert AS, Maurer DP, Haslwanter D, Sakharkar M, et al. Broad neutralization of SARS-related viruses by human monoclonal antibodies. Science. 2020;369(6504):731-736.
298. Gottlieb, R. L., Nirula, A., Chen, P., Boscia, J., Heller, B., Morris, J., ... & Skovronsky, D. M. (2021). Effect of bamlanivimab as monotherapy or in combination with etesevimab on viral load in patients with mild to moderate COVID-19: a randomized clinical trial. Jama, 325(7), 632-644.
299. Tuccori, M., Ferraro, S., Convertino, I., Cappello, E., Valdiserra, G., Blandizzi, C., ... & Focosi, D. (2020, January). Anti-SARS-CoV-2 neutralizing monoclonal antibodies: clinical pipeline. In Mabs (Vol. 12, No. 1, p. 1854149). Taylor & Francis.
300. Gu, C., Cao, X., Wang, Z., Hu, X., Yao, Y., Zhou, Y., ... & Deng, S. J. (2021). A human antibody with blocking activity to RBD proteins of multiple SARS-CoV-2 variants including B. 1.351 showed potent prophylactic and therapeutic efficacy against SARS-CoV-2 in rhesus macaques. bioRxiv.
301. Schepens, B., van Schie, L., Nerinckx, W., Roose, K., Van Breedam, W., Fijalkowska, D., ... & Saelens, X. (2021). Drug development of an affinity enhanced, broadly neutralizing heavy chain-only antibody that restricts SARS-CoV-2 in rodents. bioRxiv.
302. Koenig, P. A., Das, H., Liu, H., Kümmerer, B. M., Gohr, F. N., Jenster, L. M., ... & Schmidt, F. I. (2021). Structure-guided multivalent nanobodies block SARS-CoV-2 infection and suppress mutational escape. Science, 371(6530).
303. Ma, H., Zeng, W., Meng, X., Huang, X., Yang, Y., Zhao, D., & Jin, T. (2021). Potent Neutralization of SARS-CoV-2 by Hetero-bivalent Alpaca Nanobodies Targeting the Spike Receptor-Binding Domain. Journal of Virology.
304. Wu, X., Cheng, L., Fu, M., Huang, B., Zhu, L., Xu, S., ... & Wu, Z. (2021). A potent bispecific nanobody protects hACE2 mice against SARS-CoV-2 infection via intranasal administration. bioRxiv.
305. Yamane, D., Lu, I., Tiahjono, W., Rubidoux, L., Hussain, A., Cancilla, J. C., ... & Wang, J. (2021). Single-Domain SARS-CoV-2 S1 and RBD Antibodies Isolated from Immunized Llama Effectively Bind Targets of the Wuhan, UK, and South African Strains in vitro. bioRxiv.
306. Xu, J., Xu, K., Jung, S. K., Conte, A., Lieberman, J., Muecksch, F., ... & Casellas, R. (2021). Multimeric nanobodies from camelid engineered mice and llamas potently neutralize SARS-CoV-2 variants. bioRxiv.
307. Lu, Q., Zhang, Z., Li, H., Zhong, K., Zhao, Q., Wang, Z., ... & Tong, A. (2021). Development of multivalent nanobodies blocking SARS-CoV-2 infection by targeting RBD of spike protein. Journal of nanobiotechnology, 19(1), 1-12.
308. Singh, S., Dahiya, S., Singh, Y. J., Beeton, K., Jain, A., Sarkar, R., ... & Sehrawat, S. (2021). Targeting conserved viral virulence determinants by single domain antibodies to block SARS-CoV2 infectivity. bioRxiv.
309. Chen, X., Gentili, M., Hacohen, N., & Regev, A. (2020). A cell-free antibody engineering platform rapidly generates SARS-CoV-2 neutralizing antibodies. *bioRxiv*.
310. Armstrong, L. A., Lange, S. M., de Cesare, V., Matthews, S. P., Nirujogi, R. S., Cole, I., ... & Kulathu, Y. (2020). Characterization of protease activity of Nsp3 from SARS-CoV-2 and its in vitro inhibition by nanobodies. *bioRxiv*.
311. Wellner, A., McMahon, C., Gilman, M. S., Clements, J. R., Clark, S., Nguyen, K. M., ... & Liu, C. C. (2020). Rapid generation of potent antibodies by autonomous hypermutation in yeast. *bioRxiv*.
312. Ahmad, J., Jiang, J., Boyd, L. F., Natarajan, K., & Margulies, D. H. (2021). Synthetic nanobody–SARS-CoV-2 receptor-binding domain structures identify distinct epitopes. bioRxiv.
